# On the Nature of Fluorescence Modification Induced by Deformation in Regenerated Silk Fibroin

**DOI:** 10.1021/acsabm.5c01392

**Published:** 2025-09-25

**Authors:** Gonzalo Santoro, Óscar Toledano, Iván Horcajo Peribáñez, Esther Rebollar, Tiberio A. Ezquerra, Jose Sena-Fernández, Eduardo Solano, Mari Cruz García-Gutiérrez

**Affiliations:** † Instituto de Estructura de la Materia, IEM-CSIC, Serrano 121, 28006 Madrid, Spain; ‡ Depto. Física Interdisciplinar, Universidad Nacional de Educación a Distancia, Fac. Ciencias Avenue de Esparta s/n, Las Rozas, 28232 Madrid, Spain; § Instituto de Química Física Blas Cabrera, IQF-CSIC, Serrano 119, 28006 Madrid, Spain; ∥ ALBA Synchrotron, NCD-SWEET Beamline, Carrer de la Llum 2-26, Cerdanyola del Vallès, 08290 Barcelona, Spain; ⊥ NANOesMAT, UNED, Unidad Asociada al CSIC por el IEM y el IQF, Avenue de Esparta s/n, Las Rozas, 28232 Madrid, Spain

**Keywords:** silk fibroin, fluorescence, X-ray scattering, mechanical properties, synchrotron light, ab initio calculations

## Abstract

The mechanical deformation of regenerated silk fibroin (RSF) films induces notable changes in their fluorescence properties, yet the underlying molecular mechanisms remain poorly understood. In this work, we investigate the interplay between mechanical, structural, and optical response of RSF films processed with glycerol (gly) and with polyethylene glycol (PEG) as a plasticizer. PEG-containing films exhibited enhanced ductility, a significant increase of the absorption coefficient in the UV spectral range and narrower fluorescence spectra, with reduced intensity at longer wavelengths, suggesting fewer cross-links. These differences in the mechanical and optical properties of the investigated polymers may be due to the higher amorphous phase content of the samples with PEG, as it is revealed by wide-angle X-ray scattering (WAXS). Ab initio calculations of model systems confirmed that fluorescence, primarily due to tryptophan residues, is highly sensitive to local conformation and hydrogen-bonding interactions. To elucidate the effect of deformation on silk fibroin fluorescence, WAXS patterns, using synchrotron radiation, and fluorescence spectra were acquired while simultaneous tensile measurements were performed. Notably, strain-induced backbone alignment favors the formation of electronically coupled cross-links, which give rise to the observed changes in fluorescence. These findings provide further insights into the structure–property relationships governing RSF fluorescence, with potential implications for the design of deformation-responsive biomaterials.

## Graphical abstract

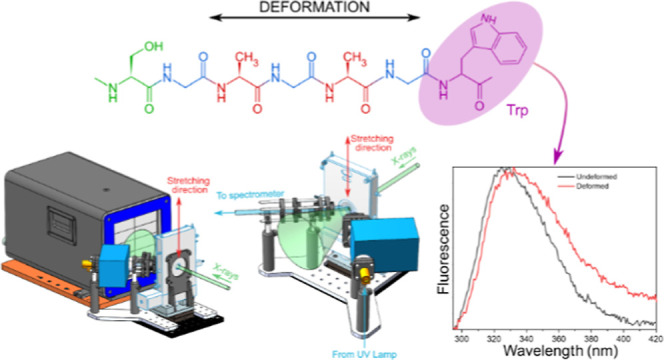

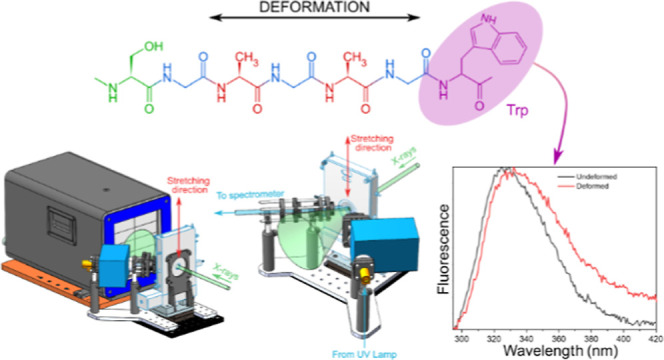

## 1 Introduction

Silk fibroin (SF), the primary structural protein produced by the silkworm *Bombyx mori*, has long been recognized for its exceptional mechanical robustness, thermal stability, biocompatibility, and environmental responsiveness.^1^ These unique properties have enabled its widespread use in biomedical applications,^2^ such as tissue engineering scaffolds, drug delivery systems, and optical biosensors.^3,4^ In particular, regenerated silk fibroin (RSF), obtained through controlled dissolution and reassembly of native silk fibers, presents a versatile platform for tuning material characteristics across a range of physical and chemical conditions.^5,6^

Among its multifunctional features, the intrinsic fluorescence of RSF has attracted increasing interest as a noninvasive, label-free probe for monitoring molecular structure, conformational changes, and degradation pathways.^4,7,8^ Unlike conventional fluorophores, RSF exhibits autofluorescence that is sensitive to environmental stimuli and structural rearrangements, providing a valuable optical signature of the protein’s state. Although some studies have reported correlations between fluorescence emission and β-sheet content,^9^ the fundamental mechanisms underlying the photophysical response of silk fibroin, particularly under mechanical stress, remain poorly understood.

Mechanical deformation is known to induce molecular reorganization of both, the crystalline and the amorphous phase as well as their interconnections, within silk fibroin.^10–12^ However, a comprehensive understanding of how such reorganization influences fluorescence emission is still lacking.

To address this gap, a multimodal strategy is required, integrating mechanical testing, fluorescence spectroscopy, structural analysis and advanced computational modeling. In this work, we performed multimodal experiments combining in situ tensile tests, fluorescence spectroscopy and synchrotron X-ray scattering, in order to investigate the fluorescence behavior of RSF films subjected to uniaxial tensile deformation, correlating emission characteristics with structural evolution. In addition, ab initio density functional theory (DFT) calculations are used to elucidate the effects of different molecular environments on the absorption and emission processes of fibroin components.

Our findings provide new insights into the opto-mechanical coupling mechanisms of fluorescence modulation induced by mechanical stress in RSF. These results not only contribute to the fundamental understanding of silk fibroin’s photophysics but also lay the groundwork for the development of sustainable deformation-responsive optical biopolymers for sensing and smart devices.

## 2 Materials and Methods

### 2.1 Materials

Soluble lyophilized silk fibroin (SF) with *M*_w_ ≥ 200 kDa, obtained from degummed *B. mori* silkworm, was purchased from Matexcel (Shirley, NY). Details on the degumming protocol are reported in the Supporting Information. Formic acid 96% (FA) was purchased from Sigma-Aldrich, glycerol 99.9% (gly) was purchased from Quimipur, and polyethylene glycol with a molecular weight of 300 g/mol (PEG) was also purchased from Sigma-Aldrich.

### 2.2 Sample Preparation

SF solutions, either in distilled water or FA, were prepared with a concentration of 60 mg/mL. These solutions were blended with gly and PEG and the mixtures were continuously stirred at 100 rpm for 1 h. The mixed solutions SF/gly/PEG were cast on 27 × 27 mm^2^ polystyrene boxes and stored at room temperature (20–25 °C) for solvent evaporation. The blending conditions as well as the film drying times are shown in Table 1. Pure SF and SF/gly films were also prepared under the same conditions.

**Table 1:** Sample Names, Preparation Conditions and Thicknesses of SF Films

samples	SF/gly/PEG (mass ratio)	drying time (days)	thickness (μm)
SF_H2O_	10:0:0	1	84 ± 5
SF_FA_	10:0:0	1	95 ± 6
SF_H2O_G	10:7:0	1	107 ± 7
SF_FA_G	10:8:0	1	116 ± 5
SF_H2O_GP	10:3:5	2	137 ± 6
SF_FA_GP	10:3:5	2	140 ± 5

### 2.3 Ultraviolet–Visible Spectroscopy

Absorption spectra of the samples used in this study, prepared as thin films spin-coated on quartz substrates, were recorded in transmission geometry using a UV–Vis spectrophotometer (UV-3600 Shimadzu, Japan). Absorption coefficient values have been derived from absorbance by normalization to the thickness of each sample film.

### 2.4 Fluorescence Spectroscopy

The fluorescence spectra of free-standing film samples were obtained using a fluorescence spectrophotometer (FluoroMax-4, USA). The fluorescence was excited at 277 nm and recorded in the wavelength range of 290–520 nm. The excitation and emission slits were set at 5 and 1 nm, respectively.

### 2.5 Mechanical Properties

The tensile properties of the specimens were measured using a Linkam TST350 stretcher cell (Linkam, UK). The distance between grips and test speeds were set at 8 mm and 0.004 mm/s, respectively. The specimens (20 mm in length × 3 mm in width) were prepared using a cutting blade. Note that sample thicknesses are listed in Table 1.

### 2.6 Set-Up for Simultaneous Tensile Test, Fluorescence and X-ray Scattering Measurements

In situ monitoring of fluorescence changes induced by deformation was carried out by using synchrotron radiation at the NCD-SWEET beamline of the ALBA synchrotron (Cerdanyola del Vallès, Barcelona, Spain). 2D wide-angle X-ray scattering (WAXS) patterns and fluorescence spectra were concurrently acquired while simultaneous tensile measurements were performed. A scheme of the setup is shown in Figure 1 and Figure S1 presents pictures of the setup installed at the NCD-SWEET beamline.

**Figure 1:**
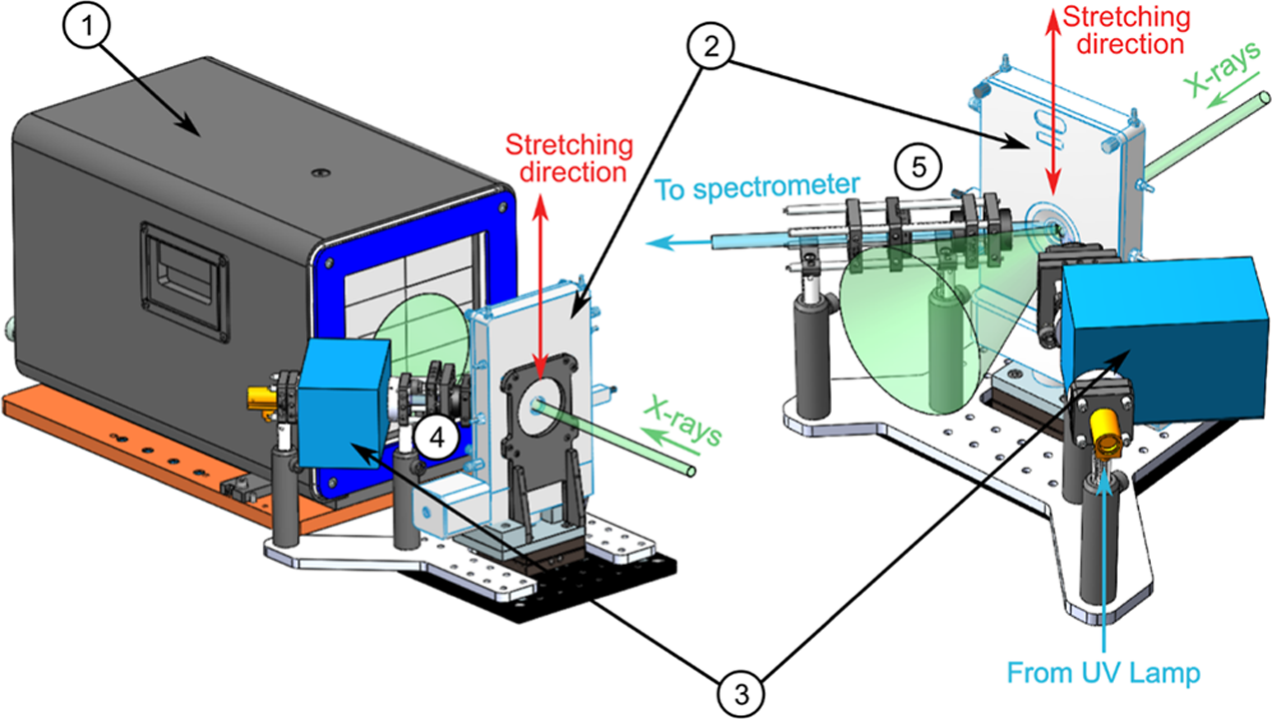
Technical drawings of the experimental setup used for the in situ experiments at the NCD-SWEET beamline. 1: X-ray detector; 2: uniaxial stretching device (the stretching direction is vertical); 3: monochromator; 4: excitation optics; 5: collection optics.

The sample is mounted vertically in the Linkam stretcher cell and perpendicular to the X-ray beam. The fluorescence spectroscopy setup, was inserted laterally between the stretcher cell and the X-ray detector (Figure 1), in order to collect the fluorescence spectra while sample stretching. In order to follow in situ, the fluorescence changes and structure modification induced by deformation, the stretcher cell, fluorescence and WAXS acquisition were simultaneously activated. Details on the instrumentation used, the data collection, data analysis and tests on radiation damage (Figure S2) are reported in the Supporting Information.

### 2.7 Ab Initio Calculations

The absorption and emission spectra of different residue sequences that are present in the silk fibroin were obtained using the Time Dependent Density Functional Perturbational Theory (TD-DFPT)^13^ as implemented in the cp2k simulation package.^14–16^ A Gaussian basis set with triple-Z radial contributions per orbital and one polarized function was used and the inner electrons were simulated using the pseudopotential approximation.^17^ To describe the exchange–correlation interaction, the PBE functional^18^ was selected, and additionally, the dispersion interactions were considered by including the Grimme D3 correction.^19^ The interaction between periodic images was minimized by including a vacuum region of 10 Å in all directions. To obtain the absorption spectra, an initial structural relaxation process was performed on each of the proposed peptide sequences, leading to an equilibrium configuration where the forces acting on every atom are below 1 × 10^–4^ a.u. (Figure S3). For these configurations, the absorption spectra were computed using the TD-DFPT formalism, from which the excitation energies and their associated oscillator strengths can be obtained.^14,15^ As each of the absorption bands leads to a different excited state, which will eventually produce a radiative decay, the emission energies and their corresponding oscillator strengths were also computed. To do so, for each excited state, which is initially in the ground state geometry, a structural relaxation was performed^20^ (Figure S3). Then, for the equilibrium configurations of each excited state, the excitation energy and the oscillator strength of the corresponding excited state were computed to obtain its emission energy and intensity. Additionally, to discuss which functional groups are involved in these absorption processes, the excited state and ground state electron densities were also represented.

## 3 Results and Discussion

### 3.1 Mechanical Properties of Silk Fibroin Blend Films

To elucidate the effect of different solvents and additives on silk fibroin mechanical properties, tensile measurements were carried out. The stress–strain curves for SF_H2O_G, SF_FA_G, SF_H2O_GP and SF_FA_GP are shown in Figure S4. Three samples of every composition were tested. The curves corresponding to the SF_H2O_ and SF_FA_ samples are not presented because, given the fragility of these samples, it was not possible to cut them without breaking them. It is noticeable that the elongation at break of SF films increases significantly with the addition of PEG, while the Young modulus decreases. In addition, it is observed that samples dissolved in water present higher Young modulus than those dissolved in formic acid (Figure 2).

**Figure 2:**
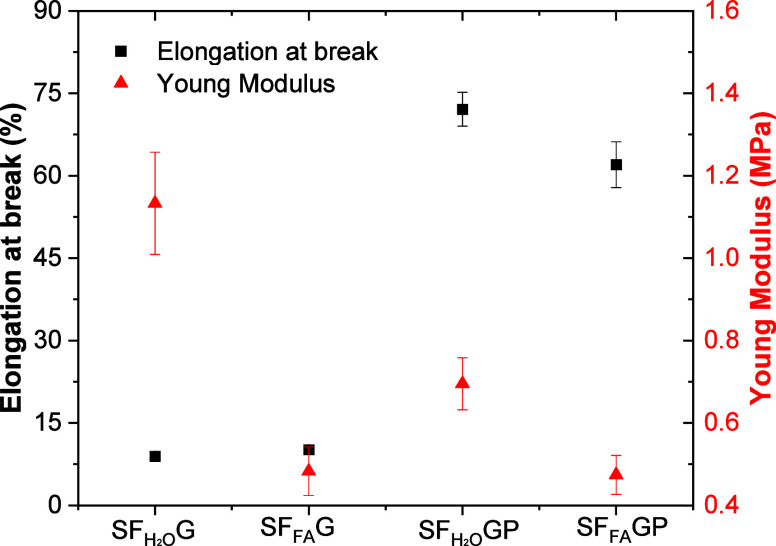
Elongation at break and Young modulus of SF samples with different solvents and additives, as indicated.

### 3.2 Optical Properties of Silk Fibroin Blend Films

The absorption coefficients of SF_H2O_G, SF_FA_G, SF_H2O_GP and SF_FA_GP, as a function of wavelength, are shown in Figure 3a. In general trends, the absorption coefficient is higher for samples dissolved in water than for those dissolved in formic acid, even for plain SF (Figure S5a). In addition, the absorption coefficient increases significantly with the addition of PEG. The films can absorb UV light in the spectral range of 200–300 nm. The well-defined peak in the 250–300 nm region is attributed to aromatic amino acids such as tyrosine, phenylalanine and tryptophan, which are present in the fibroin sequence;^21^ this peak has its maximum at 277 nm, related to tryptophan absorption.^22,23^ In the visible range, the absorbance was negligible for all the investigated materials.

**Figure 3:**
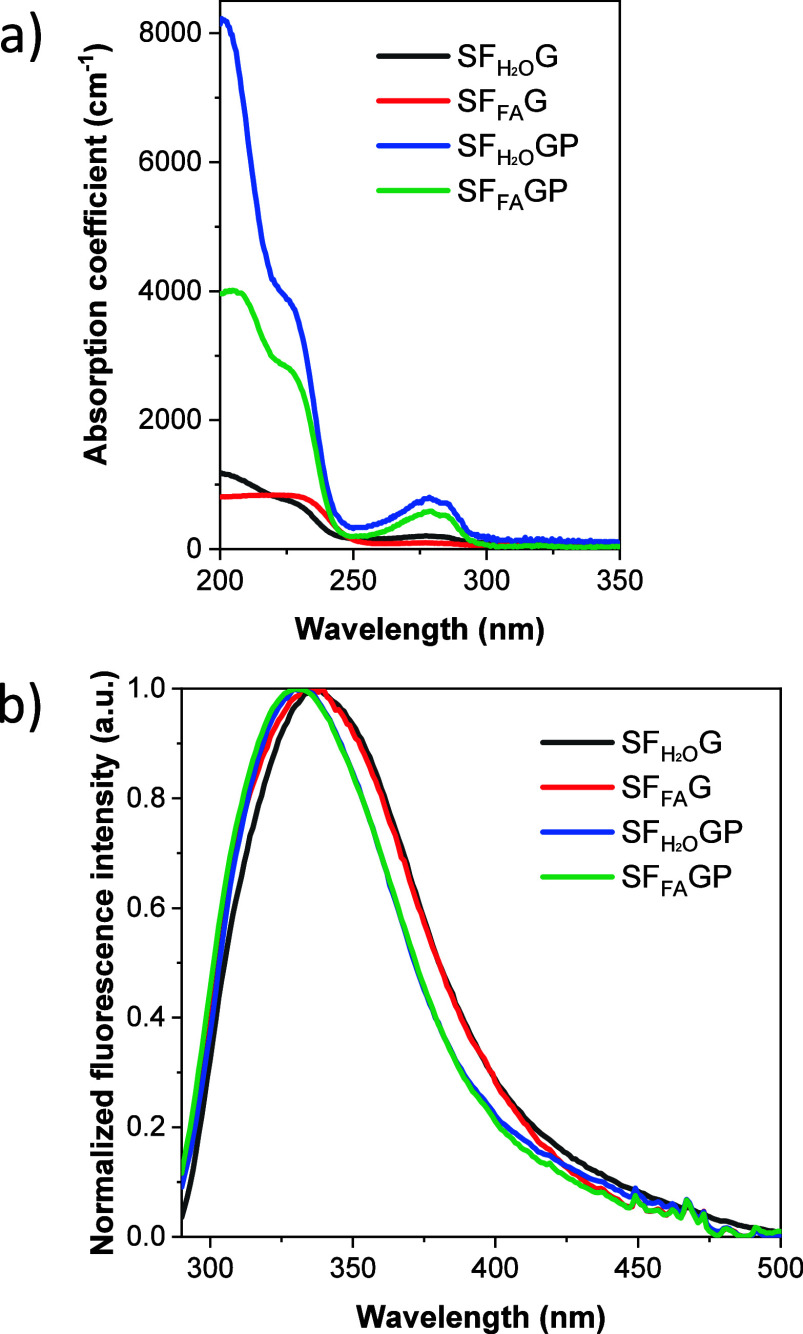
(a) Absorption coefficients and (b) normalized fluorescence emission spectra (excitation at 277 nm) of the investigated samples, as indicated.

Figure 3b shows the normalized fluorescence spectra acquired with 277 nm excitation from SF_H2O_G, SF_FA_G, SF_H2O_GP and SF_FA_GP films. Similar spectra are observed for silk fibroin samples SF_H2O_ and SF_FA_ (Figure S5b). The broad fluorescence peak relates to tryptophan, tyrosine and cross-links, being this last contribution located at longer wavelengths.^9^ In addition, the peak maximum at around 330 nm is characteristic of tryptophan.^4,9,23,24^ It has been also published by Vivian and Callis^25^ that the position of the peak maximum associated with tryptophan is considerably sensitive to its local environment, ranging from ∼308 nm to ∼355 nm. It is observed in Figure 3b that the samples with PEG show narrower fluorescence spectra, losing weight the contribution at longer wavelengths. This contribution, as it was mentioned before, is attributed to cross-links.^9^

### 3.3 Fluorescence Changes Induced by Deformation

To elucidate the effect of deformation on silk fibroin fluorescence emission, fluorescence spectra were acquired while simultaneous tensile measurements were performed, as indicated in the methods section. Figure 4a shows representative normalized fluorescence emission spectra, acquired with 277 nm excitation, from SF_H2O_GP films before sample stretching and after 60% of strain. These spectra exhibit significant differences. First, a shift of the maximum of the fluorescence band to longer wavelengths is observed in the deformed sample as compared to the undeformed one. Another clear difference between both spectra in Figure 4a is a broadening of the fluorescence spectrum as the sample is stretched, indicating an increase in the contribution at longer wavelengths which, as indicated above, corresponds to cross-links. A similar behavior is observed in the fluorescence spectrum of the SF_FA_GP sample when stretched (Figure S6c). In the case of the SF_H2O_G and SF_FA_G samples, due to their brittleness, they cannot be stretched enough to observe any changes. (Figure S6a,b). The time evolution of the fluorescence maxima as a function of strain is shown in Figure 4b. It is observed that the shift of the fluorescence maximum to longer wavelengths starts after about 18.5% of strain. The time evolution of the fluorescence maxima as a function of strain for samples SF_H2O_G, SF_FA_G and SF_FA_GP is presented in Figure S7. We measured the fluorescence as a function of strain of identical samples obtaining similar results.

**Figure 4:**
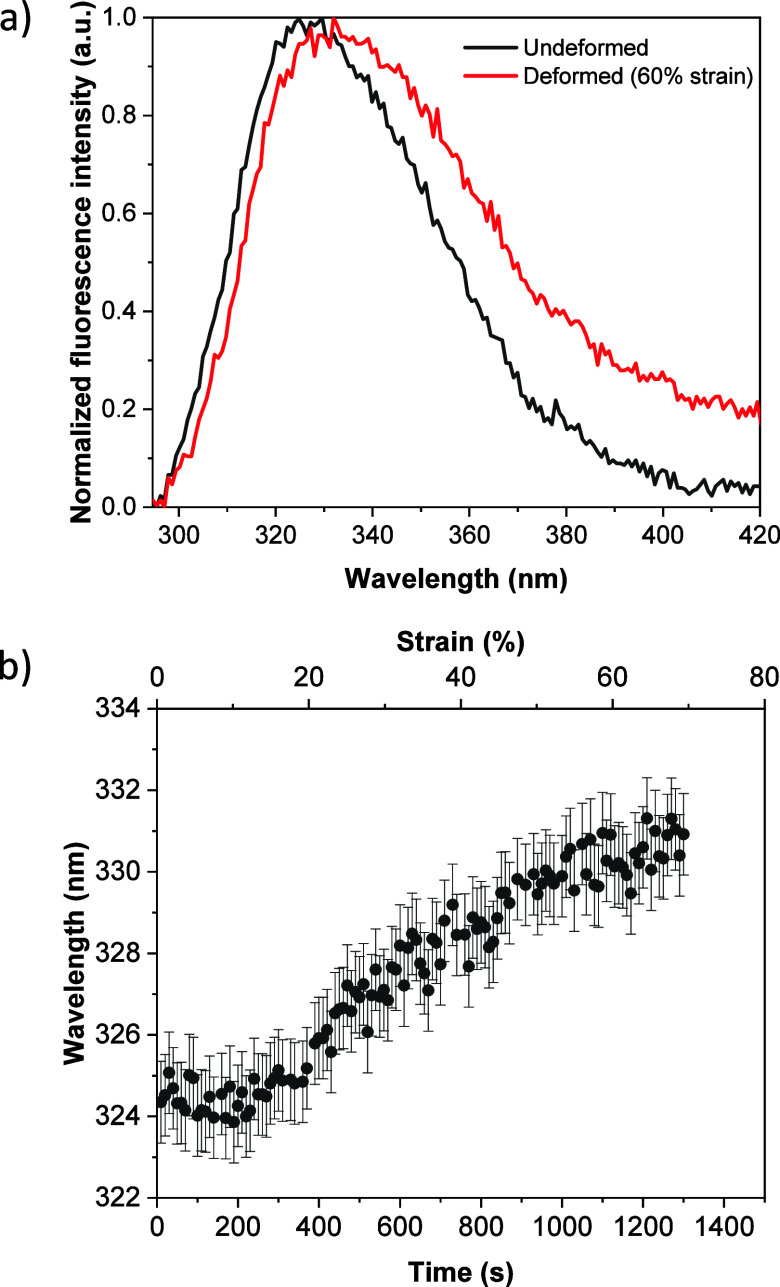
(a) Normalized fluorescence spectra (excitation at 277 nm) of SF_H2O_GP before and during sample stretching, as indicated. (b) Real time evolution of the fluorescence maxima as a function of strain.

### 3.4 Secondary Structure-Properties Relationship

The secondary structures of SF_H2O_G, SF_FA_G, SF_H2O_GP and SF_FA_GP films were investigated by wide-angle X-ray scattering (WAXS), to elucidate the relationship between properties and the silk fibroin secondary structure. Figure 5a shows the reduced WAXS I­(q) profiles of the SF_H2O_G, SF_FA_G, SF_H2O_GP and SF_FA_GP films, extracted from the 2D patterns (Figure 5b) by azimuthally integration over 360°, as shown in Figure S8a.

**Figure 5:**
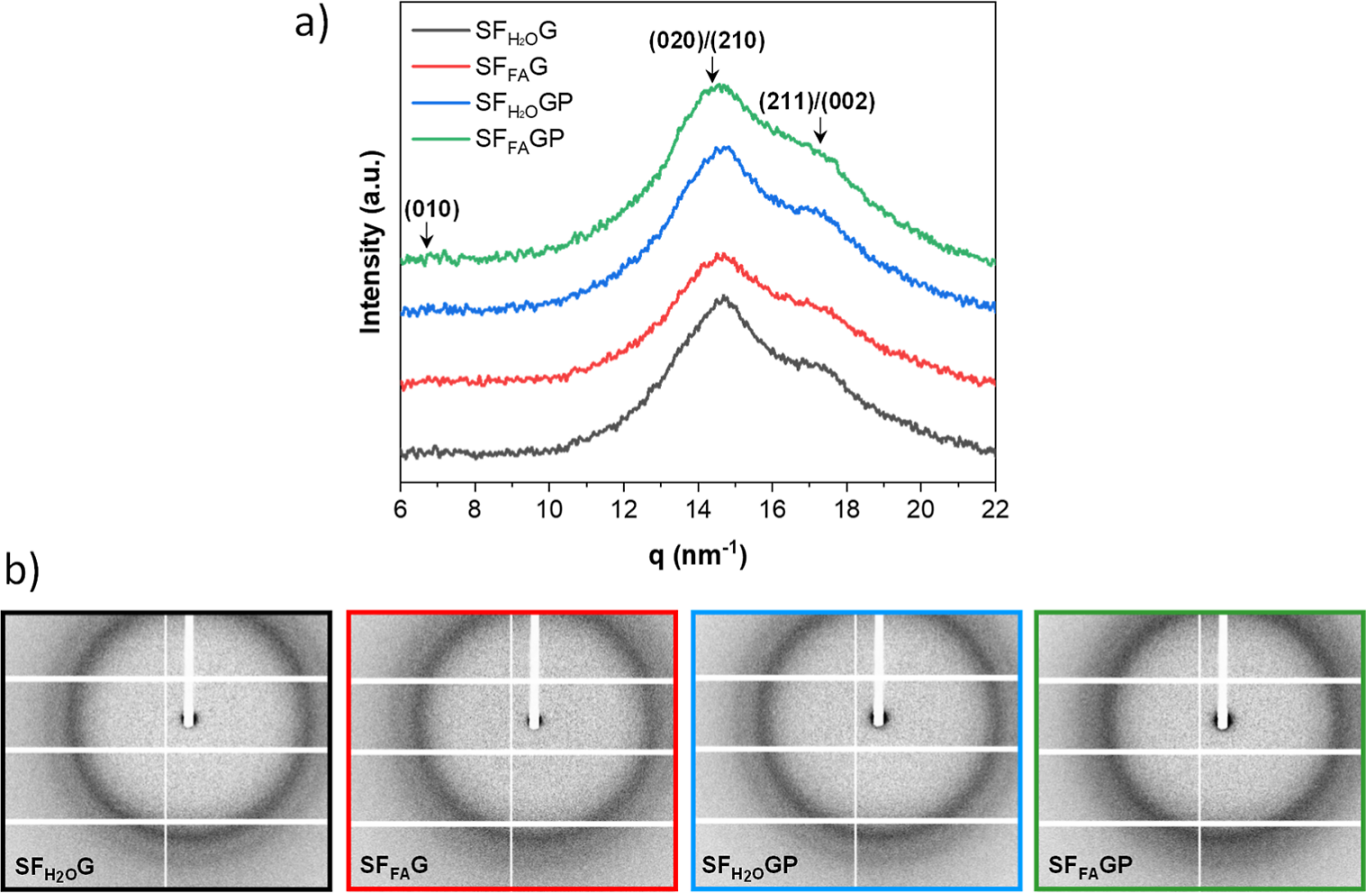
(a) WAXS 1-D intensity profiles of the investigated samples, as indicated. The profiles have been shifted vertically for clarity. The positions of selected reflections are indicated. (b) 2D WAXS patterns after background subtraction.

The crystallinity of the different samples was calculated from the area of the crystal peaks divided by the total area of the crystal peaks and the amorphous halo, by fitting the 1-D intensity profiles using Gaussian curves (Figure S8b,c). No crystal features from PEG and glycerol were observed. The assignment of the Bragg reflections corresponding to the silk II structure, shown in Figures 5a and S8b,c, was carried out according to the literature.^26–29^ The crystallinity of SF_H2O_G, SF_FA_G, SF_H2O_GP and SF_FA_GP films is shown in Figure 6. It is observed that although all samples present crystallinity values in the range from 22 to 29%, in agreement with literature,^28^ the lower values correspond to samples with PEG. This indicates an increase in the amorphous content due to the plasticizer effect of PEG. In Section 3.1 it was pointed out that the elongation at break of SF films increases significantly with the addition of PEG, therefore we could correlate the more ductile behavior of SF_H2O_GP and SF_FA_GP with the increase of amorphous content. Concerning the microstructure-fluorescence relationship for the investigated samples, it has been pointed out (Figure 3b) that the samples with PEG show narrower fluorescence spectra, losing weight the contribution at longer wavelengths, attributed to cross-links. Our hypothesis is that the cross-links, mainly related to hydrogen bonds as it will be extensively discussed in Section 3.5, are more favored in the crystal silk II structure (rich in β-sheet) than in the amorphous state.

**Figure 6:**
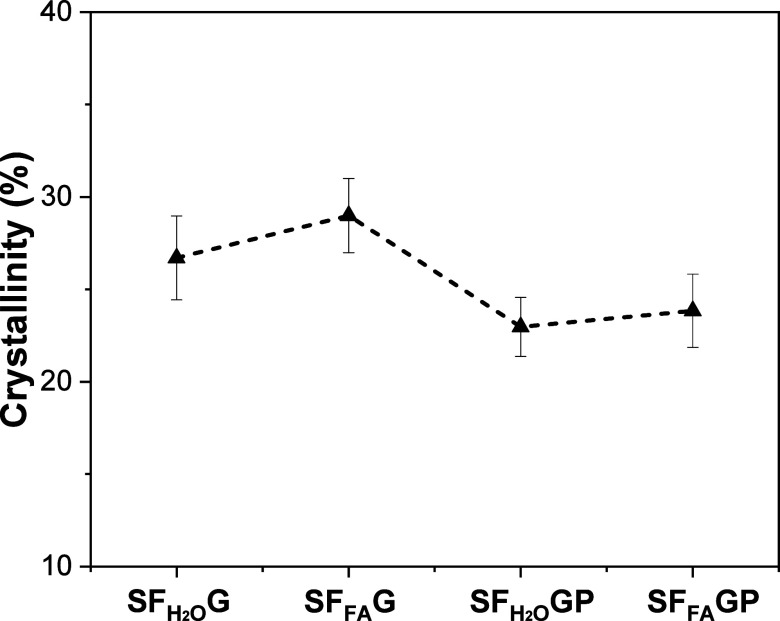
Crystallinity of the investigated SF samples determined through the fitting of their WAXS 1-D intensity profiles.

The evolution of the structural content of SF_H2O_G, SF_FA_G, SF_H2O_GP and SF_FA_GP films was investigated in real time as a function of deformation. Figure 7 shows the crystallinity of SF_H2O_GP as a function of strain and time. It is interesting to note that the crystallinity increases slightly as increasing strain. A similar behavior is observed for SF_H2O_G, SF_FA_G and SF_FA_GP samples when stretched (Figure S9). In this case we cannot explain the effect of deformation on silk fibroin fluorescence considering only the increase of crystallinity with increasing strain. This is so because for samples SF_H2O_GP and SF_FA_GP the increase of cross-links contribution at high wavelengths with strain is evident (see Figures 4a and S6c) while for samples SF_H2O_G and SF_FA_G it is negligible (see Figure S6a,b).

**Figure 7:**
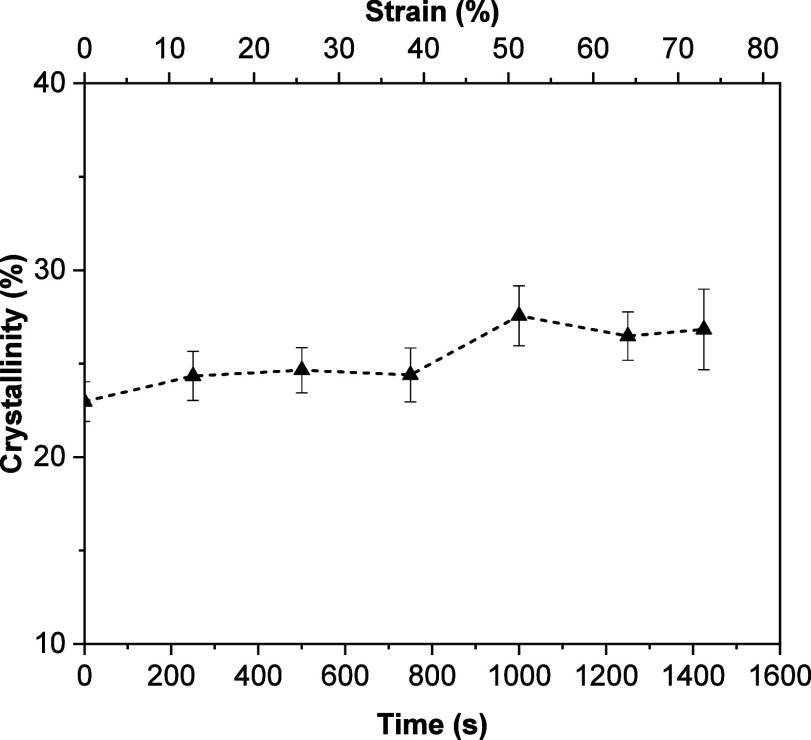
Real time evolution of the crystallinity of SF_H2O_GP, determined by fitting the WAXS 1-D intensity profiles, as a function of strain.

#### 3.4.1 Molecular Orientation

In order to understand the main reasons behind the changes in silk fibroin fluorescence during deformation, we have performed a quantitative analysis of the 2D WAXS patterns collected in situ, during sample deformation, in terms of molecular orientation. The oriented fraction parameter, Φ, and the Hermans orientation function, *f*_2_, have been estimated. The oriented fraction quantifies the amount of crystals preferentially oriented in a certain direction, while the Hermans orientation function is the degree of orientation with respect to that direction. The procedure to calculate these magnitudes has been explained in detail in previous works.^30–34^ Details on the strategy to estimate the oriented fraction and the Hermans orientation function from the 2D WAXS patterns are reported in the Supporting Information.

Figure 8a,b shows the values of the oriented fraction and of the Hermans orientation function, respectively, as a function of time and strain. It is observed that the oriented fraction increases with increasing strain, while the orientation function tends to the value *f*_2_ = −0.5 with increasing strain, indicating that the scattered intensity of reflection (020) concentrates on the equator. Considering the geometry of our experiment (see Figure 1) and that the *b* axis of the unit cell is parallel to the axis of the polypeptide chains and the *a* and *c* axes lie in the basal plane perpendicular to the *b* axis,^35,36^ then the axis of the polypeptide chains corresponds to the stretching direction. A similar behavior is observed for the SF_FA_GP sample when stretched (Figure S11e,f). In the case of the SF_H2O_G and SF_FA_G samples, due to their brittleness, they cannot be stretched enough to observe a clear trend with deformation (Figure S11a–d). Therefore, we hypothesize that the orientation of the polypeptide chains along the stretching direction may induce the formation of new cross-links which contribute to the broadening of the fluorescence spectrum as the sample is stretched. This is supported by the quantum mechanical calculations.

**Figure 8:**
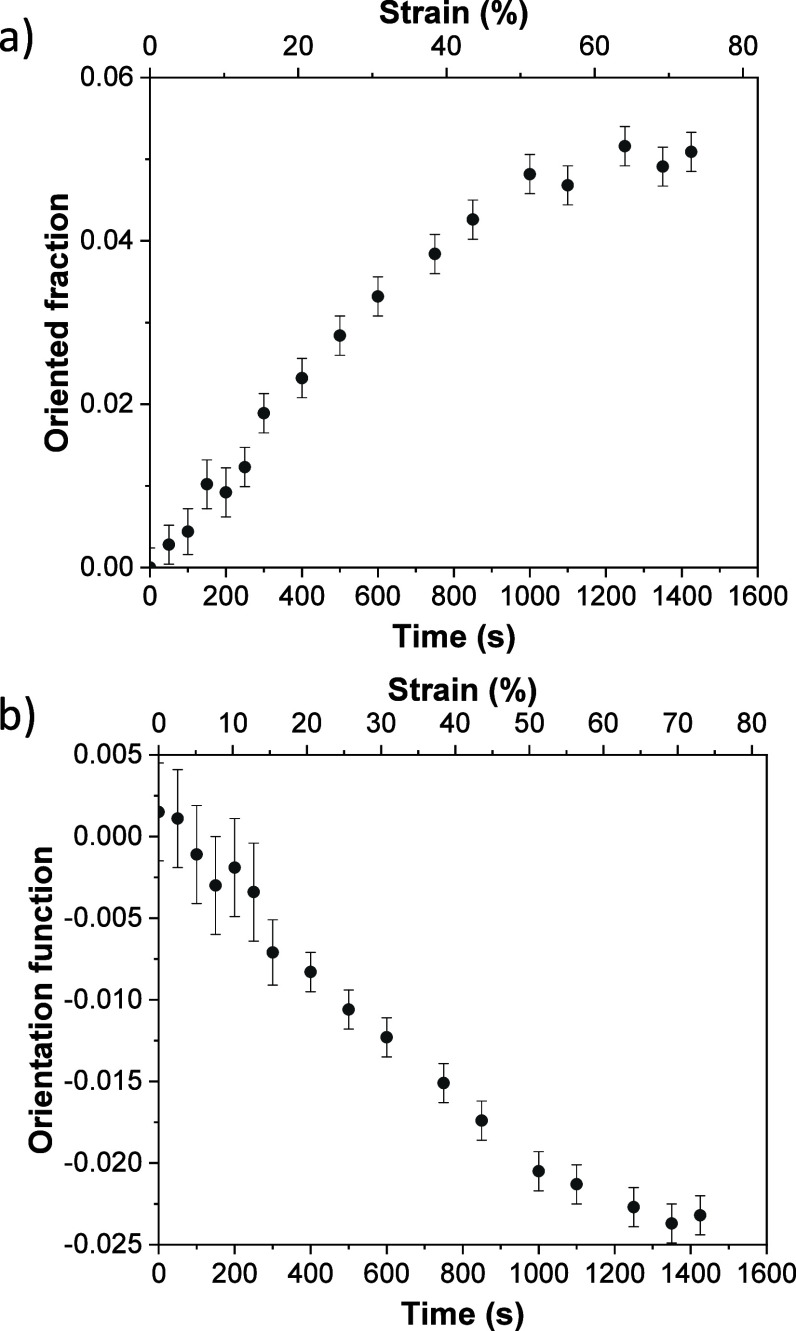
Real time evolution of (a) the oriented fraction and (b) the Hermans orientation function of SF_H2O_GP, obtained from the 2D WAXS patterns, as a function of strain.

### 3.5 Quantum Mechanical Calculations

To gain insight into the change in fluorescence upon silk fibroin deformation, we performed ab initio quantum mechanical calculations. Both Tryptophane (Trp) and Tyrosine (Tyr) residues are known to be responsible for the fluorescence in most of the proteins,^37^ and particularly in silk fibroin.^38^ However, the contribution of Tyr to silk fibroin fluorescence is only residual for solid material.^38^ In addition, it has been experimentally demonstrated that the absorption/emission spectra of Tyr are practically insensitive to its chemical environment.^37^ Therefore, we have focused on the Trp residue as it might be responsible for the observed changes in the fluorescence.

Due to the complexity of silk fibroin, we calculated the absorption and emission spectra of model systems based on a basic unit (GTrp) composed of Glycine (Gly) bonded to Trp (Figure 9a). Glycine represents more than 40% of the molar composition of the silk fibroin.^39,40^ Although Alanine is also present in considerable amount (about 30% of silk fibroin composition^39,40^ ), Gly has been selected for simplicity. This particular selection does not affect the results presented here as the important H-bonding interactions between Trp and the silk fibroin backbone involve the NH_2_ and COOH groups. The effect of the CH_3_ moiety of Alanine will be minimal, if any.

**Figure 9:**
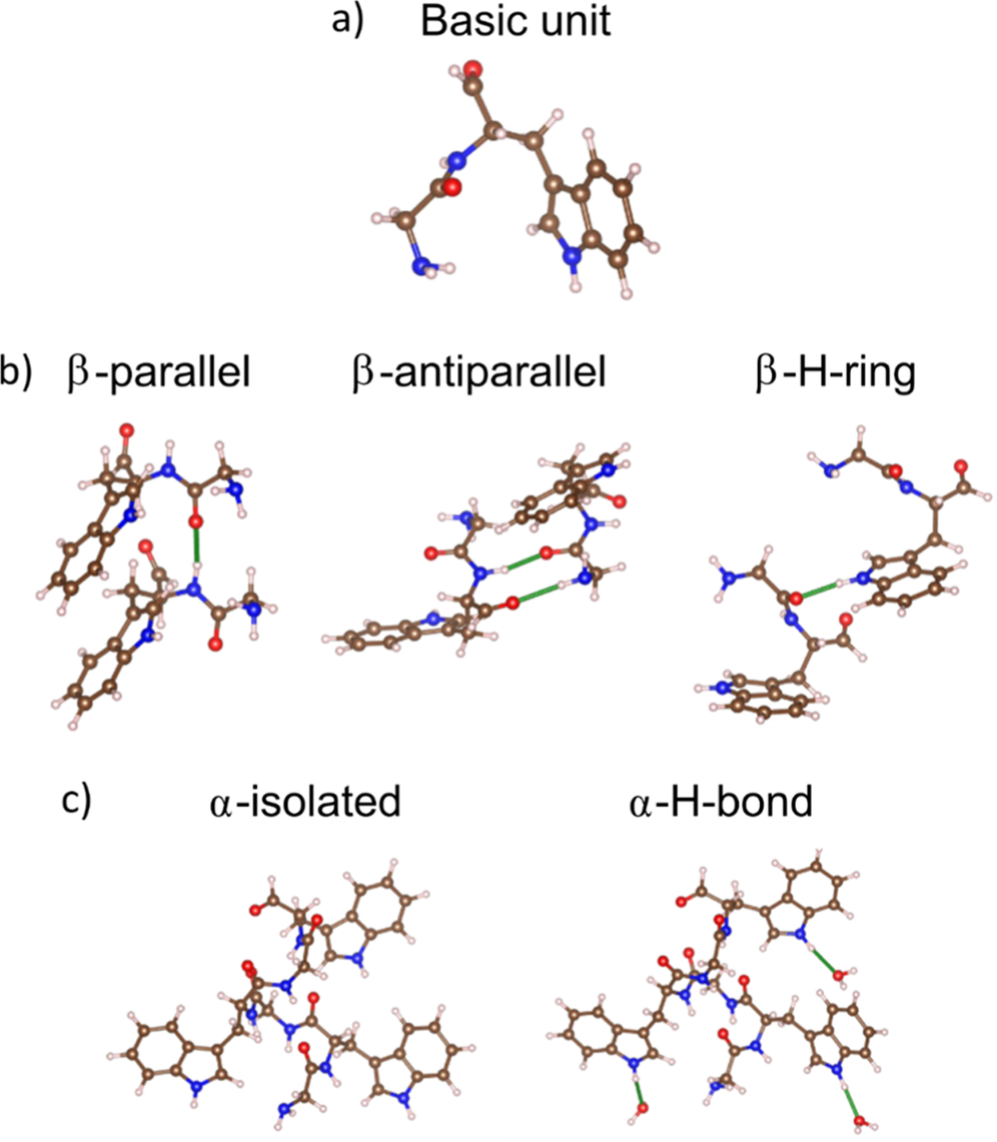
Model systems used for the ab initio calculations. (a) basic molecular unit; (b) molecular structures based on β-sheet arrangements; (c) molecular structures associated with α-helix configurations.

We have explored six different structures. The first one is the basic unit (Figure 9a). Three molecular structures composed of two basic units: β-parallel, β-antiparallel and β-H-ring. These structures account for the different H-bonding configurations in β-sheet arrangements (Figures 9b and S12a). β-Parallel and β-antiparallel consist in the typical parallel and antiparallel β-sheet disposition, respectively (hydrogen bonds are formed between the –NH and –CO groups). In the β-H-ring the indole group of the Trp residue forms a hydrogen bond with the –CO of the Gly moiety. Finally, we considered two additional structures: α-isolated and α-H-bond. Both are associated with α-helix configurations (Figure 9c and S12b) using three basic units with G-Trp-G-Trp-G-Trp sequence (see Figure 9c). The α-H-bond structure is surrounded by H_2_O molecules forming hydrogen bonds with the –NH group of the indole rings of the Trp residue. This last structure was considered to account for the effect of H-bonding but it is not representative of our material.

We note that, although the molecular configurations we have chosen are compatible with β and α structures, these are not related to crystal structures. Only the different H-bonding configurations are explored and no crystalline structure has been considered. We also note that our calculations present some limitations as we only consider small peptide models due to the complexity and computational costs of using full silk fibroin structures. In addition, the calculations do not consider solvent effects since our materials are in solid form.

The total absorption spectra, which include all the non-negligible excitation transitions, are presented in Figure 10. As we used an excitation wavelength of 277 nm in the experiments, we have studied in more detail the absorption transitions within the 250–300 nm range. In particular, we have focused on the absorption transitions leading to nonvanishing emission (see Figure S3 for a schematic representation of the computational methodology employed). These absorption bands along with their associated emissions are also presented in Figure 10 and summarized in Table 2.

**Figure 10:**
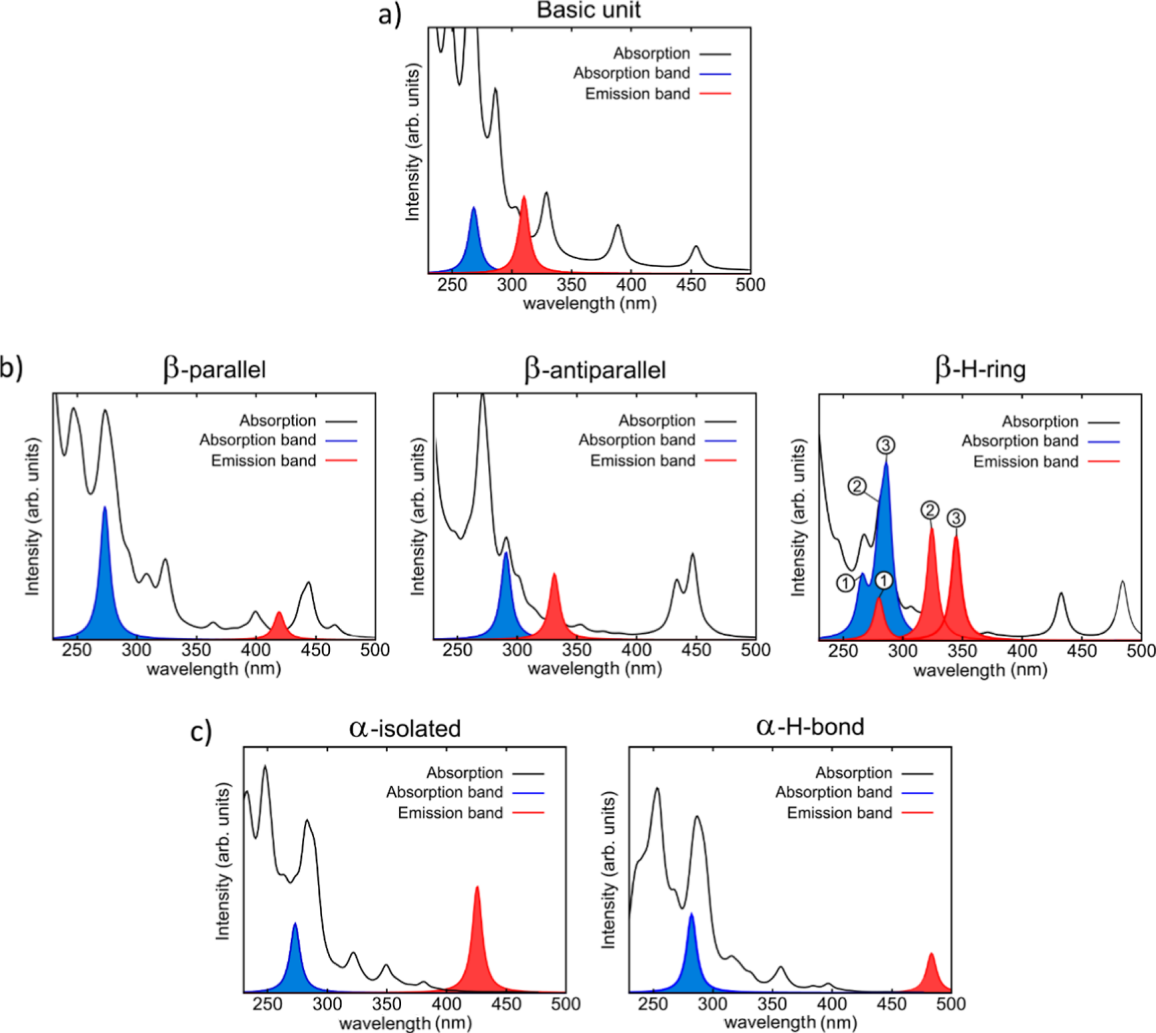
Absorption and emission spectra of the different configurations of the GTrp residue: (a) basic molecular unit; (b) molecular structures based on β-sheet arrangements; (c) molecular structures associated with α-helix configurations. Black: total absorption spectra. Blue: absorption bands that after structural relaxation of the excited states lead to emission with nonvanishing oscillator strength. Red: emission bands associated with the absorption transitions. In (b) labels 1,2 and 3 indicate the relationship within the absorption and emission peaks, i.e., the emission labeled as 1 is associated with the absorption labeled as 1 and so on.

**Table 2:** Calculated Wavelengths for the Main Absorption and Emission Bands of Molecular Model Systems Shown in Figure 10

structure	absorption bands λ(nm)	emission bands λ(nm)
Basic unit	268	310
β-parallel	273	419
β-antiparallel	291	331
β-H-ring	266, 280, 287	324, 344
α-isolated	273	419
α-H-bond	282	483

For the basic unit we found an absorption band centered at 268 nm that leads to an emission at 310 nm. The value of the Stokes shift (∼40 nm) is consistent with that observed experimentally for Trp under different solvent conditions.^37^

In the case of the three structures based on β-sheet arrangements (β-parallel, β-antiparallel and β-H-ring), the main absorption bands are red-shifted with respect to that of the basic unit. In particular, main absorptions at 273 and 291 nm are observed for the β-parallel and β-antiparallel structures, respectively. For the β-H-ring structure, three main absorption bands are centered at 266 nm, 280 and 287 nm (labeled 1, 2, and 3 in Figure 10).

More interestingly, we have observed that the emission transitions are also considerably red-shifted with respect to that of the basic unit. The main emission for β-parallel and β-antiparallel structures are observed at 419 and 331 nm, respectively, and the emission bands of β-H-ring occur at 324 and 344 nm (labeled 2 and 3 in Figure 10). These results imply that the formation of H-bonds produces a red-shift of the Trp residue fluorescence irrespective of the exact configuration of the H-bonding network. This is further confirmed when considering the absorption/emission of the α-isolated and α-H-bond structures. Both structures show larger redshifts with respect to those based on β-sheet arrangements. In the case of the α-isolated structure, the absorption at 273 nm produces an emission at 419 nm. This large redshift can be attributed to the strong coupling between the electronic states localized in the indole group and those of the nearest carbonyl and amino groups that form the hydrogen bonding network (Figures S13 and S14). More importantly, the main emission for the α-H-bond structure occurs at 483 nm for the absorption at 282 nm. Thus, the formation of H bonding by the indole group produces a remarkable red-shift of the emission peaks even if the absorption experiences a less dramatic red-shift. Here we stress again that the α-H-bond structure is not representative of our material but was considered to account for the effect of H-bonding on the absorption/emission properties.

Overall, our calculations show that the formation of an H-bonding network produces a red-shift on the fluorescence of the Trp moiety which is consistent with the experimental results. Therefore, the calculations point toward the promotion of H-bonding upon silk fibroin deformation. This is also consistent with the slight increase in crystallinity as the crystallites are stabilized by hydrogen bonds. Nevertheless, the observed changes in the fluorescence cannot be solely attributed to a change in crystallinity. A considerable fraction of H-bonding must occur in the amorphous. This is the most mobile phase and it will experience larger conformational modifications upon uniaxial deformation.

## 4 Conclusions

This study demonstrates that the fluorescence of regenerated silk fibroin films is modulated by mechanical deformation through structural rearrangements at the molecular level. The incorporation of PEG enhances chain mobility and promotes the formation of amorphous domains, resulting in more ductile material that exhibits a significant increase of the absorption coefficient in the UV spectral range and narrower fluorescence spectra, with reduced intensity at longer wavelengths, suggesting fewer cross-links. In situ monitoring of silk fibroin fluorescence changes induced by deformation was carried out by using synchrotron radiation, showing a pronounced fluorescence red-shift and spectral broadening upon strain. WAXS analyses confirmed increased chain alignment in stretched samples, particularly in PEG-containing systems. Quantum mechanical calculations revealed that the emission properties of tryptophan residues are highly dependent on local hydrogen-bond networks and secondary structure. The emergence of red-shifted emission is consistent with the formation of cross-link-like interactions between aromatic residues and the backbone under mechanical load, which are very likely promoted not only by the orientation of the crystalline phase but also by the orientation of the amorphous domains. Together, these results establish a clear link between macroscopic deformation, molecular conformation, and fluorescence behavior in RSF, paving the way for the rational design of silk-based materials with tunable mechano-optical properties. Our results present implications for future developments in biosensing, smart materials, and bioinspired design.

## Supplementary Material



## Notes

Details on the setup for simultaneous tensile test, fluorescence and X-ray scattering measurements. Pictures of the experimental setup used for the in situ experiments installed at the NCD-SWEET beamline. Tests of X-ray radiation damage. Scheme of the methodology employed to calculate the absorption and emission energies and schematic representation of the workflow followed in this study. Representative stress–strain curves for SF samples with different solvents and additives. Absorption coefficients and normalized fluorescence of plain silk fibroin dissolved in different solvents as indicated. Normalized fluorescence of SF_H2O_G, SF_FA_G and SF_FA_GP, before and during sample stretching. Real time evolution of the fluorescence maxima as a function of strain of SF_H2O_G, SF_FA_G and SF_FA_GP. Fitting of the WAXS 1-D intensity profiles of SF_H2O_G and SF_H2O_GP. Crystallinity, as a function of strain and time, of SF_H2O_G, SF_FA_G and SF_FA_GP. Details on the strategy to estimate the oriented fraction and the Hermans orientation function from the 2D WAXS patterns. Real time evolution of the oriented fraction and the Hermans orientation function as a function of strain of SF_H2O_G, SF_FA_G and SF_FA_GP. Schematic representation of the typical parallel β-sheet and α-helix configurations. Electron density of the ground and excited states involved in the absorption processes that lead, after structural relaxation, to a relevant fluorescent emission. Electron density of the ground state and excited states involved in the emission processes with relevant oscillator strengths (PDF)
