# Supplementary material for: On the Nature of Fluorescence Modification Induced by Deformation in Regenerated Silk Fibroin
Source: ACS Appl Bio Mater. 2025 Sep 25;8(10):9268–76. doi: 10.1021/acsabm.5c01392 (PMC12541700; doi:10.1021/acsabm.5c01392)
Supplement: Supplementary file 1 [file mt5c01392_si_001.pdf]

Supporting Information

On the nature of fluorescence modification induced by deformation in regenerated silk fibroin

Gonzalo Santoro[1], Óscar Toledano[1,2,+], Iván Horcajo Peribáñez[1], Esther Rebollar[3], Tiberio A. Ezquerra[1], Jose Sena-Fernández[1], Eduardo Solano[4], Mari Cruz García-Gutiérrez[1,*]

[1]Instituto de Estructura de la Materia, IEM-CSIC, Serrano 121, 28006 Madrid, Spain

[2]Universidad Nacional de Educación a Distancia (UNED), Depto. Física Interdisciplinar, Fac. Ciencias Av. de Esparta s/n, 28232, Las Rozas, Madrid, Spain.

[3]Instituto de Química Física Blas Cabrera, IQF-CSIC, Serrano 119, 28006 Madrid, Spain

[4]ALBA Synchrotron, NCD-SWEET beamline, Carrer de la Llum 2-26, Cerdanyola del Vallès, 08290, Barcelona, Spain

[+]NANOesMAT, UNED, Unidad Asociada al CSIC por el IEM y el IQF, Av. de Esparta s/n, 28232, Las Rozas, Madrid, Spain.

*Corresponding author: maricruz@iem.cfmac.csic.es

Details on the degumming protocol

Degumming Method: Hot Alkali Boiling.

Dissolution/Precipitation Method: Lithium Bromide (LiBr) Hydrolysis.

In this process, the degummed semi-finished product is dissolved in LiBr, followed by dialysis and purification to yield a clear silk fibroin solution. While uniformity testing is not conducted, consistency is optimized by meticulously controlling conditions such as temperature, concentration, and time at each stage of production.

Details on the set-up for simultaneous tensile test, fluorescence and X-ray scattering measurements

Fluorescence measurements were performed under specular reflection conditions at an incident angle of 45° using monochromatic UV light at 277 nm as excitation. This was achieved by monochromatizing the emission of a Laser-Driven Light Source (EQ-99X LDLS, Energetiq, Hamamatsu Photonics) which was subsequently focused on the sample at the same position that was probed by X-rays. We note that the UV beam size is of a few millimeters whereas the X-ray beam was 130 x 130 microns. UV-Vis collection optics were mounted at 90° with respect to the incident UV beam and the fluorescent signal was refocused at an optical fiber (QP600-2-SR-BX) coupled to the 25 μm input slit of the spectrometer. Fluorescence spectra were acquired with a grating spectrometer (Ocean Optics, model QE65000) with a 300 grooves/mm grating and a cooled linear CCD-array detector (Hamamatsu S7031-1006). The spectral range of the instrument is 200–980 nm, with 0.8 nm spectral resolution. We used an integration time of 2 s per spectrum, and fluorescence spectra were continuously acquired during the uniaxial deformation. For the WAXS measurements an X-ray wavelength (λ) of 0.124 nm was set using a Si (111) channel cut monochromator. The scattering patterns were recorded with a Pilatus3S 1M detector (981 × 1043 pixels, 172.0 × 172.0 μm^2 pixel size) which was located at 0.204 m from the sample position. A standard Cr_2O_3 (from NIST) was used to

calibrate the sample-to-detector distance, detector tilts, and reciprocal space. WAXS patterns were acquired every 5 s with a 1 s acquisition time, thus avoiding radiation damage, as can be seen in Figure S2. 2D WAXS data reduction and analysis were performed by sing the Fit2D software[1]. For background correction the patterns from air, obtained with the sample out of the beam, were subtracted from the sample patterns.

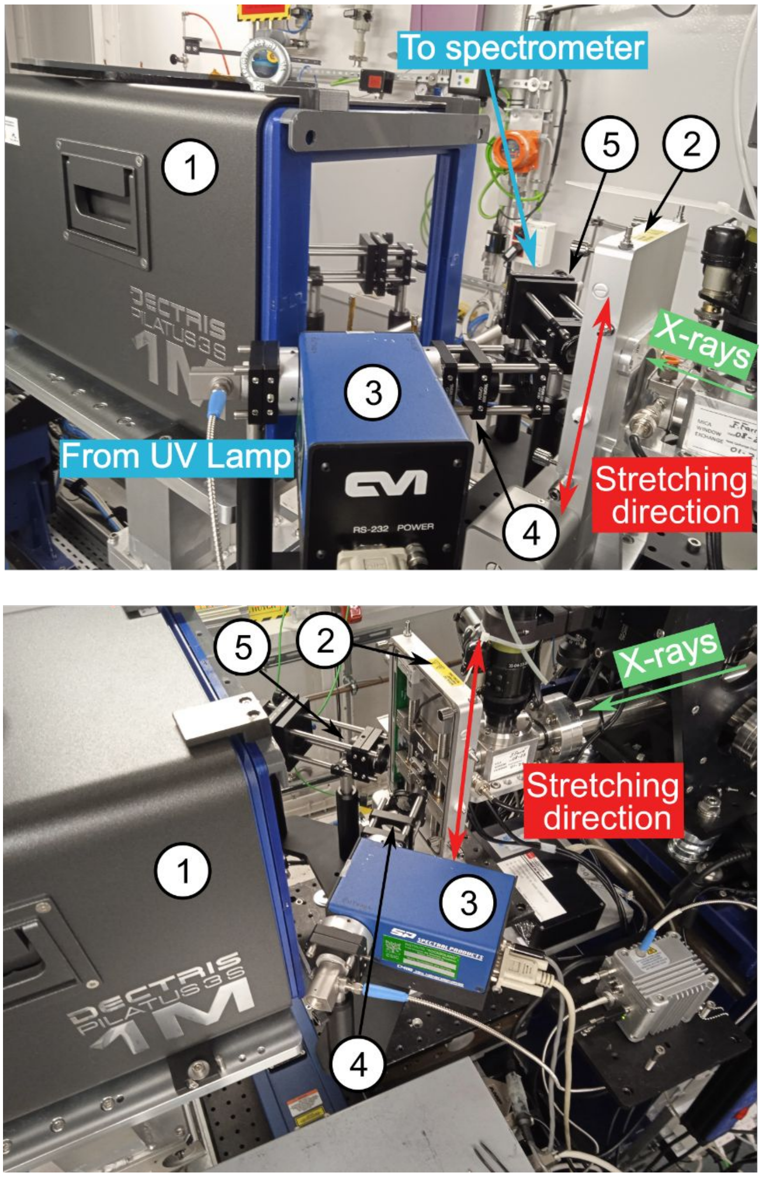

Figure S1. Pictures of the experimental setup used for the *in-situ* experiments installed at the NCD-SWEET beamline. 1: X-ray detector; 2: uniaxial stretching device (the stretching direction is vertical); 3: monochromator; 4: excitation optics; 5: collection optics.

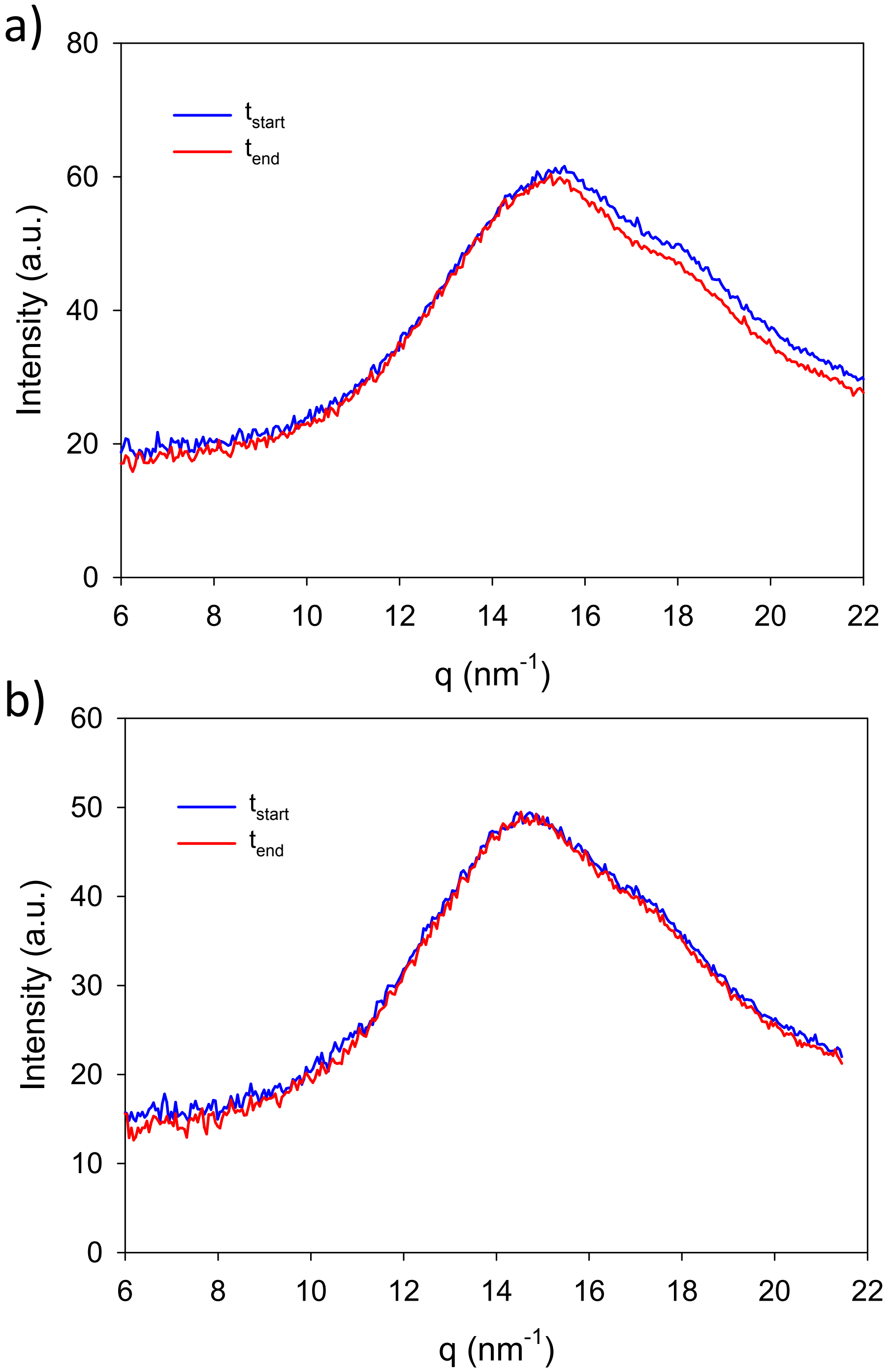

Figure S2. Tests of X-ray radiation damage. I (q) WAXS profiles at the starting time and at the final time of the experiments for a) $SF_{H2O}GP$ and b) $SF_{FA}GP$.

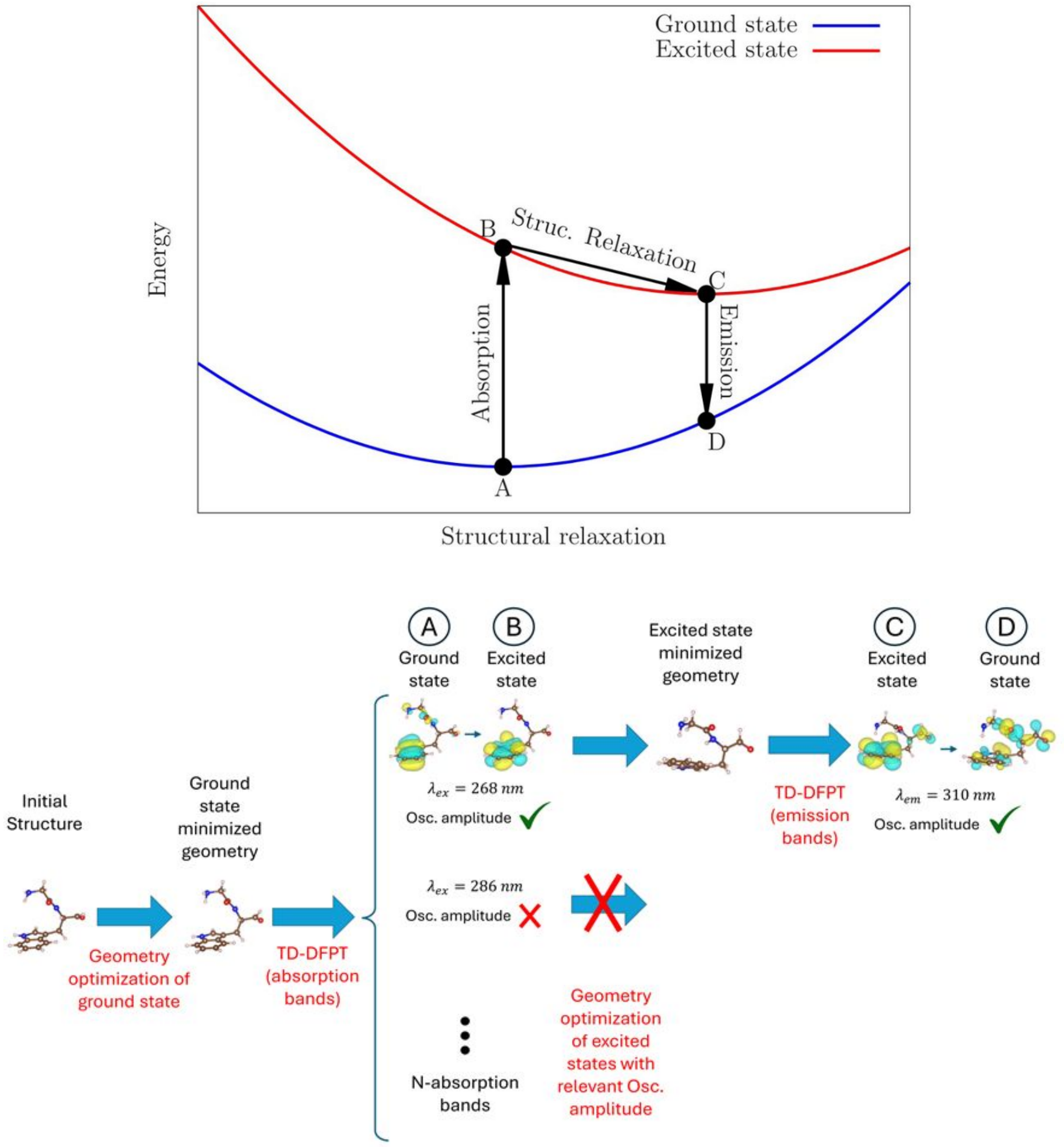

Figure S3. (Top) Scheme of the methodology employed to obtain the absorption and emission energies. First, the transition from the ground to the excited state was calculated (A-B). Then, structural relaxation was performed (B-C). Finally, the emission transition energy was calculated from the relaxed excited state (C-D). (Bottom) Schematic representation of the workflow followed in this study. For each of the proposed structures (initial structure), geometry optimization is performed to obtain the equilibrium structures (ground state minimized geometry). For these minimized structures, a TD-DFPT calculation is conducted, leading to a series of transitions (N-absorption bands) from occupied orbitals of the ground state (A) to virtual orbitals in the excited state (B), with their associated oscillator amplitudes. All transitions with negligible oscillator amplitude were discarded, as they do not contribute in a significant manner to the absorption. For those transitions with significant oscillator amplitude, the geometry of the corresponding

excited state has been optimized. Finally, for the resulting geometry, the oscillator amplitude of the emission transition from the excited state (C) to the ground state (D) has been evaluated. Only processes with non-vanishing oscillator amplitudes in both the absorption and emission transitions have been considered relevant in the fluorescence phenomena.

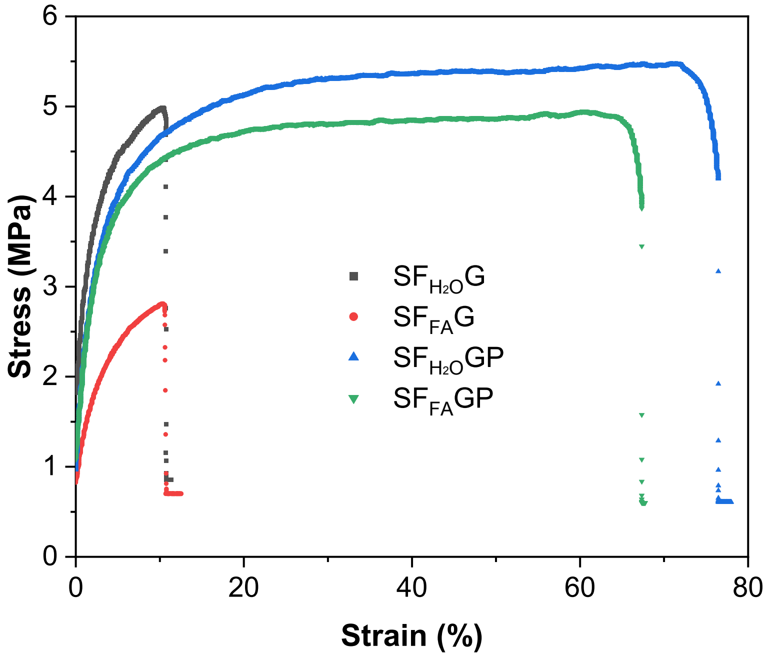

Figure S4. Representative stress-strain curves for SF samples with different solvents and additives, as indicated. The reader is referred to the Table 1 of the main text for sample description.

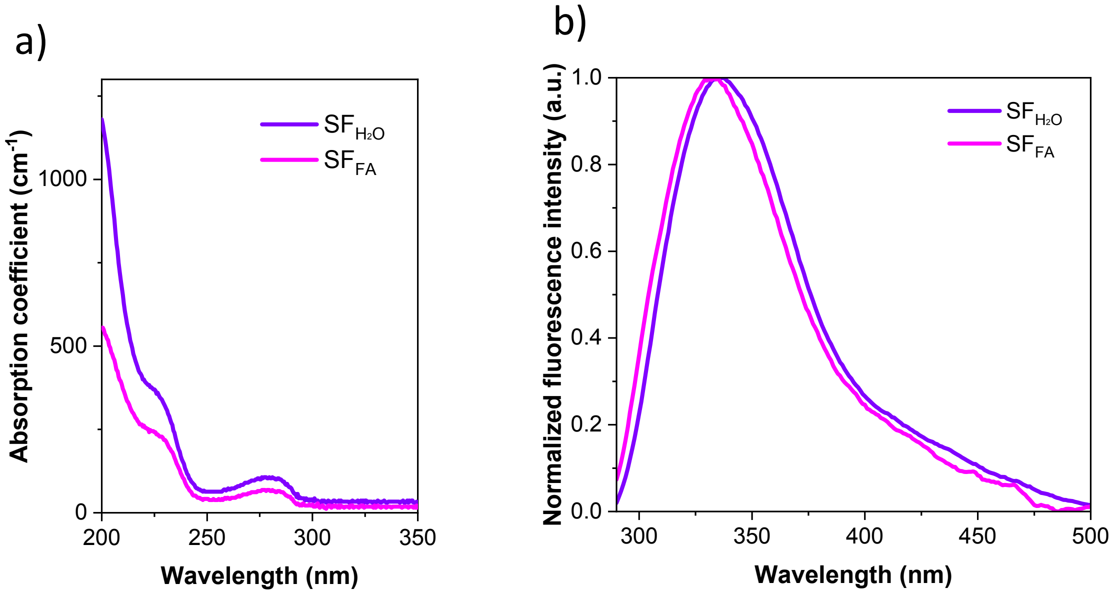

Figure S5. a) Absorption coefficients and b) normalized fluorescence emission spectra (excitation at 277 nm) of plain silk fibroin dissolved in different solvents as indicated.

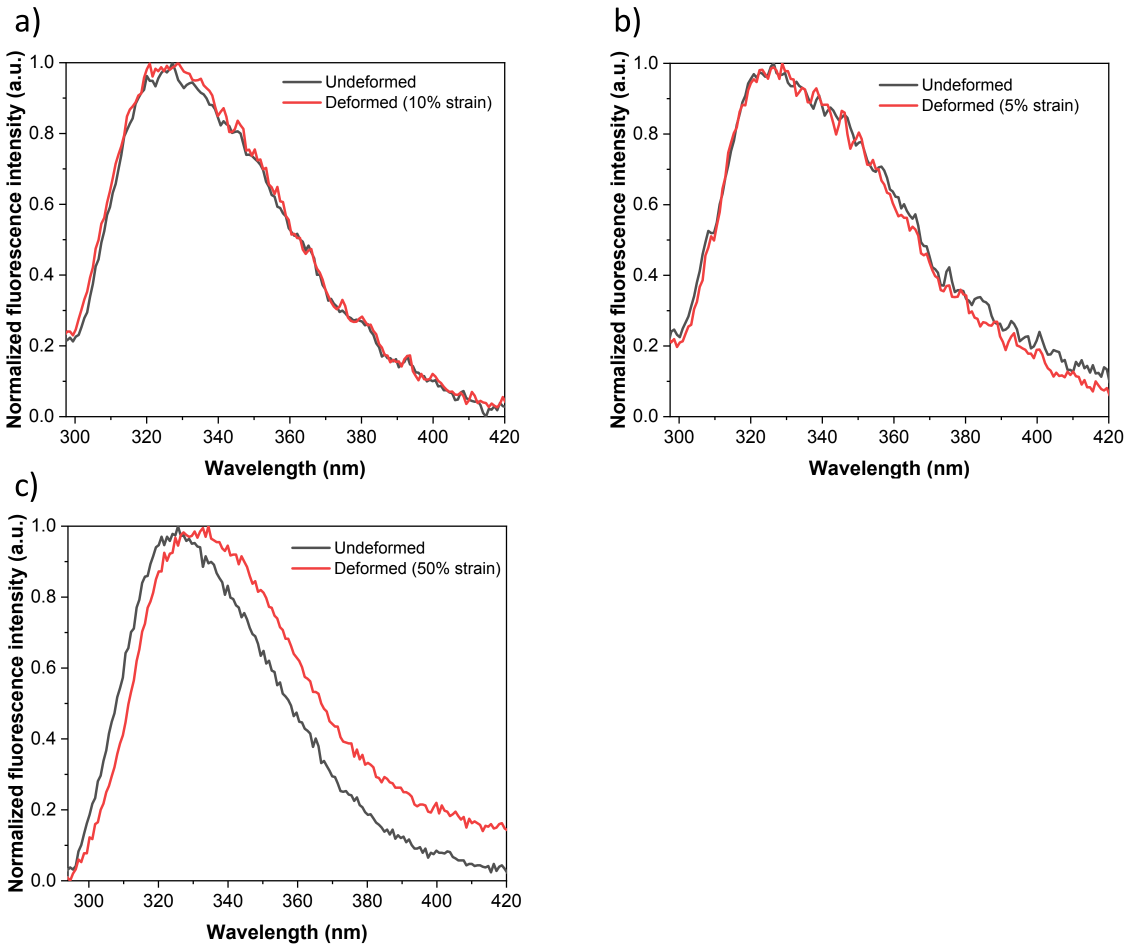

Figure S6. Normalized fluorescence emission spectra (excitation at 277 nm) of a) $SF_{H2O}G$, b) $SF_{FA}G$ and c) $SF_{FA}GP$, before and during sample stretching, as indicated.

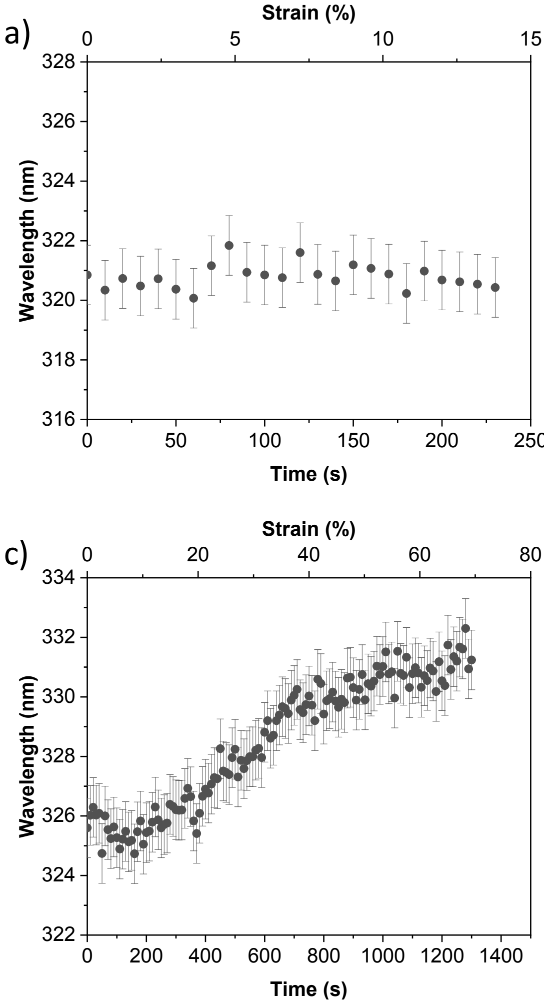

Figure S7. Real time evolution of the fluorescence maxima as a function of strain of a) $SF_{H2O}G$, b) $SF_{FA}G$ and c) $SF_{FA}GP$.

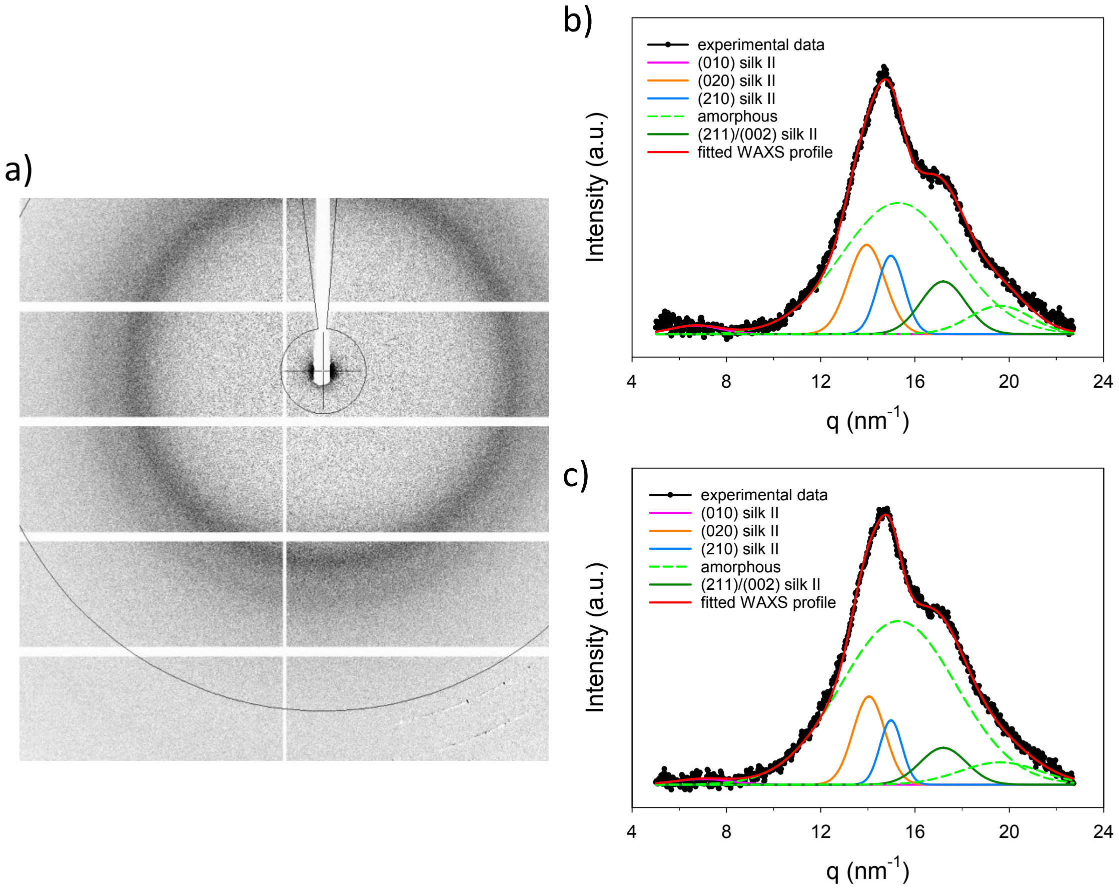

Figure S8. a) 2D WAXS pattern showing the cake used for extracting the I (q) 1-D profiles. Fitting of the WAXS 1-D intensity profiles of: b) $SF_{H2O}G$ and c) $SF_{H2O}GP$.

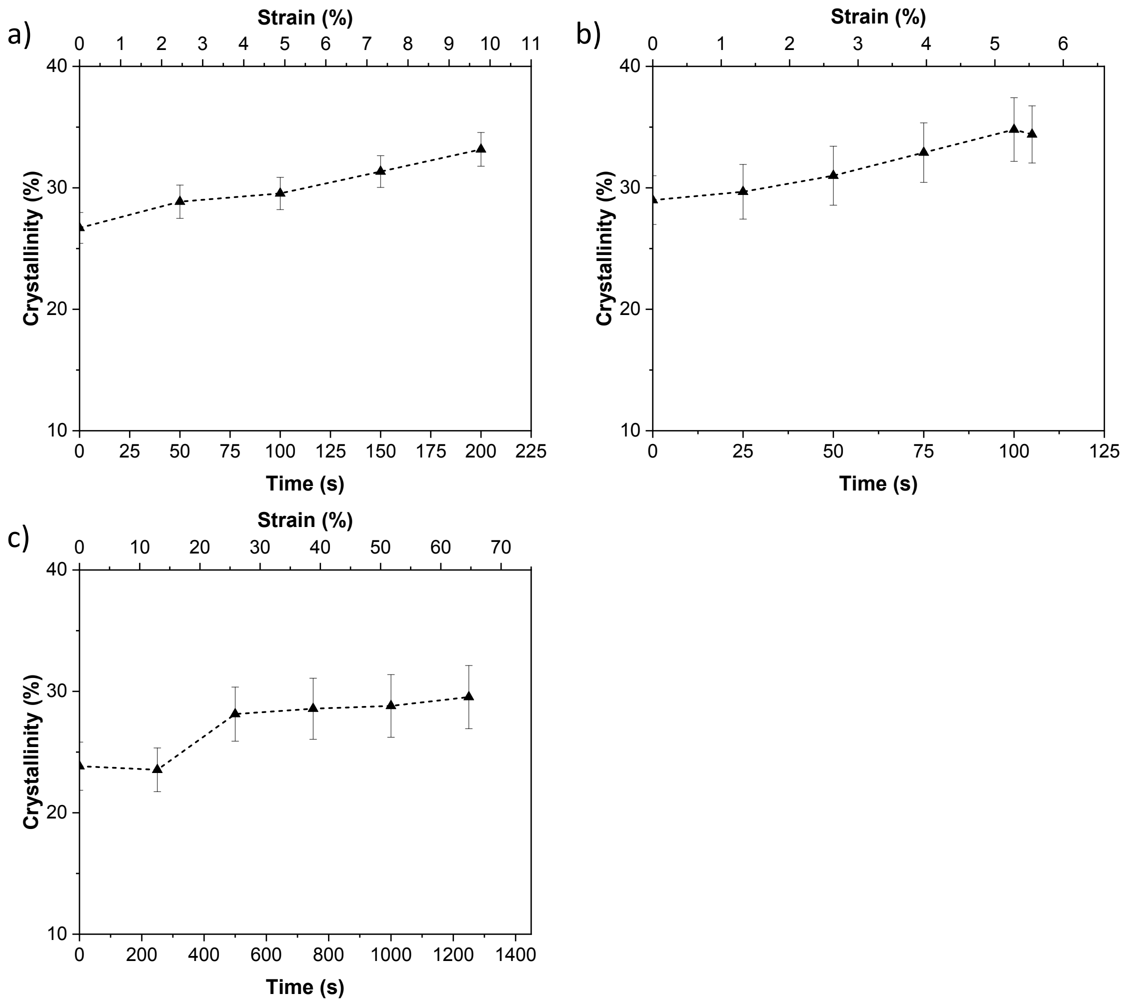

Figure S9. Crystallinity, as a function of strain and time, of: a) $SF_{H2O}G$, b) $SF_{FA}G$ and c) $SF_{FA}GP$.

Details on the strategy to estimate the oriented fraction and the Hermans orientation function from the 2D WAXS patterns

The oriented fraction can be obtained by dividing the 2D WAXS pattern into two quadrants chosen to represent the scattering centered along the meridian (0 – 180°) and the equator (90 – -90°) respectively. The procedure is illustrated in Figure S10 where two cakes, represented by the continuous lines, are defined in each quadrant with a width of 40 pixels. The cakes are centered at the q-values corresponding to the maximum scattered intensity of the (020) Bragg reflection. The scattered intensity is radially integrated within the cakes and the oriented fraction parameter Φ can be defined by means of:

$$\Phi = \frac{\mathrm{I_{mer}} - \mathrm{I_{eq}}}{\mathrm{I_{mer}} + \mathrm{I_{eq}}} \quad (1)$$

where I_{mer} and I_{eq} are the integrated scattered intensity for the meridian and equator contributions, respectively. The Hermans orientation function f_2 (the second order Legendre polynomial of $< cos^2 \varphi >$) can also be estimated from the 2D WAXS patterns and is given by:

$$f_2 = \frac{3 < cos^2 \varphi > -1}{2} \quad (2)$$

with:

$$< cos^2\varphi >= \frac{\sum_{\varphi_i=-90}^{0} I_i sin\varphi_i cos^2\varphi_i}{\sum_{\varphi_i=-90}^{0} I_i sin\varphi_i} \quad (3)$$

with I_i being the scattered intensity at the ith azimuthal angle φ_i. The zero position for φ was set at the meridian of the pattern and φ runs counterclockwise (see Figure S8). The initial and final angles for the integration were chosen to be φ = -45° and φ = 45° respectively. The orientation function takes the value f_2 = 1 when the scattered intensity

concentrates on the meridian and $f_2 = -0.5$ when the scattered intensity concentrates on the equator. For an isotropic pattern, a value of $f_2 = 0$ is expected.

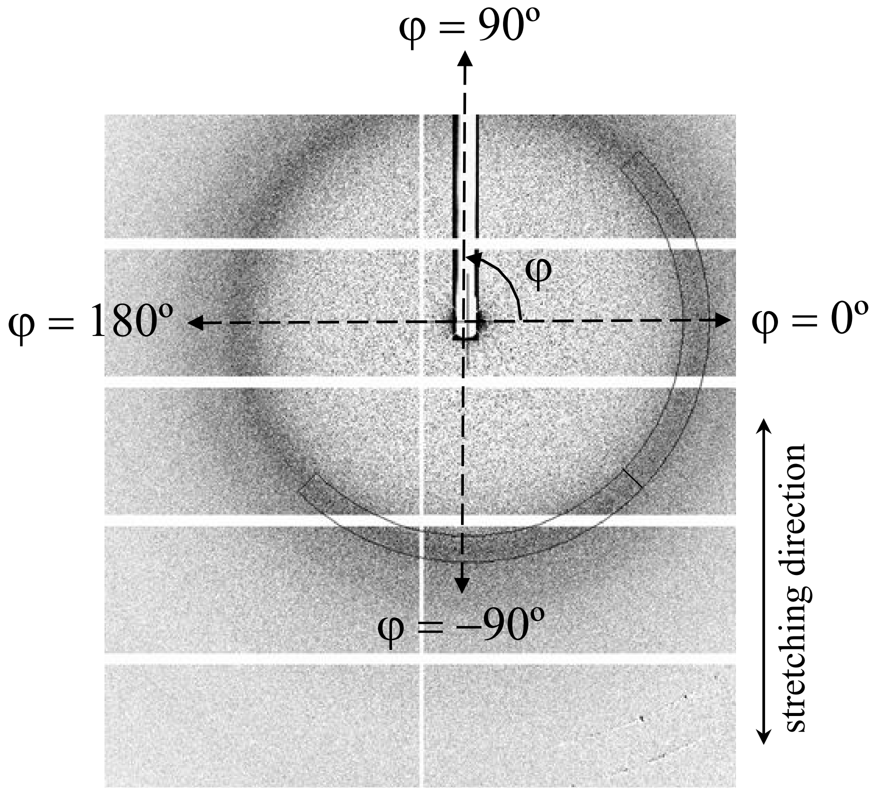

Figure S10. Strategy to estimate the oriented fraction from the 2D WAXS pattern. Two cakes, represented by the continuous lines, are defined in each quadrant, equatorial and meridian, with a width of 40 pixels. The cakes are centered at the q-values corresponding to the maximum scattered intensity of the (020) Bragg reflection. The scattered intensity is radially integrated within the cakes and from equation 1 the oriented fraction is obtained.

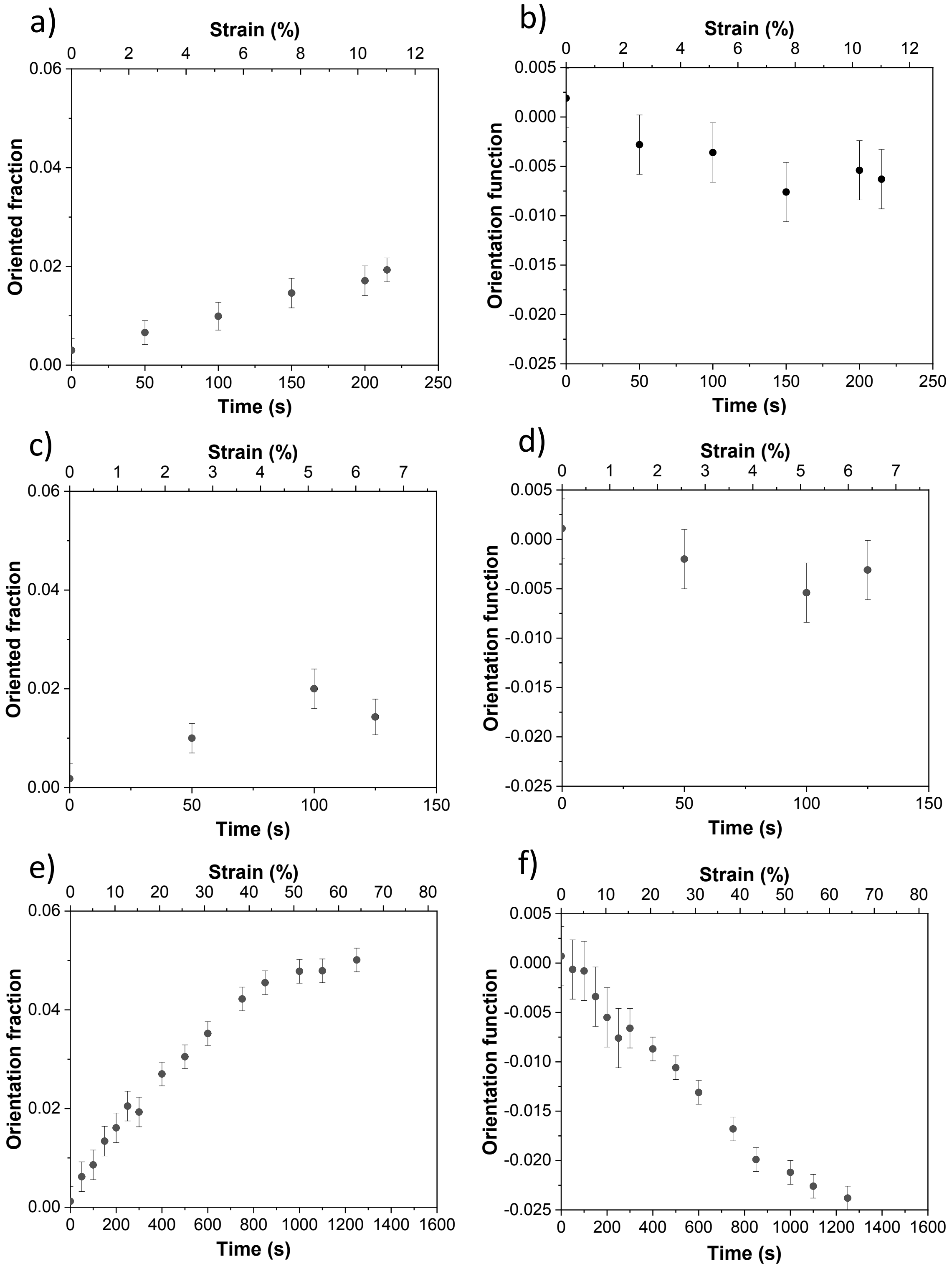

Figure S11. Real time evolution of the oriented fraction as a function of strain of a) $SF_{H2O}G$, c) $SF_{FA}G$, e) $SF_{FA}GP$, and real time evolution of the Hermans orientation function as a function of strain of b) $SF_{H2O}G$, d) $SF_{FA}G$, f) $SF_{FA}GP$.

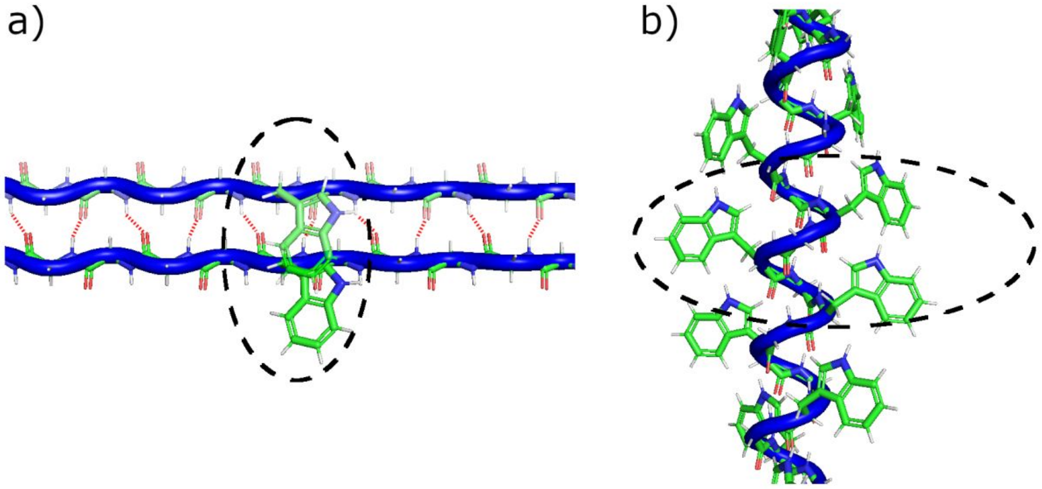

Figure S12: Schematic representation of the typical parallel β-sheet (a) and α-helix (b) configurations. The model systems used in the theoretical study are encircled with dashed black lines. In the case of the β-sheet, two G-Trp units belonging to adjacent β-sheet chains are included. In the case of the α-helix, three consecutive G-Trp units are considered.

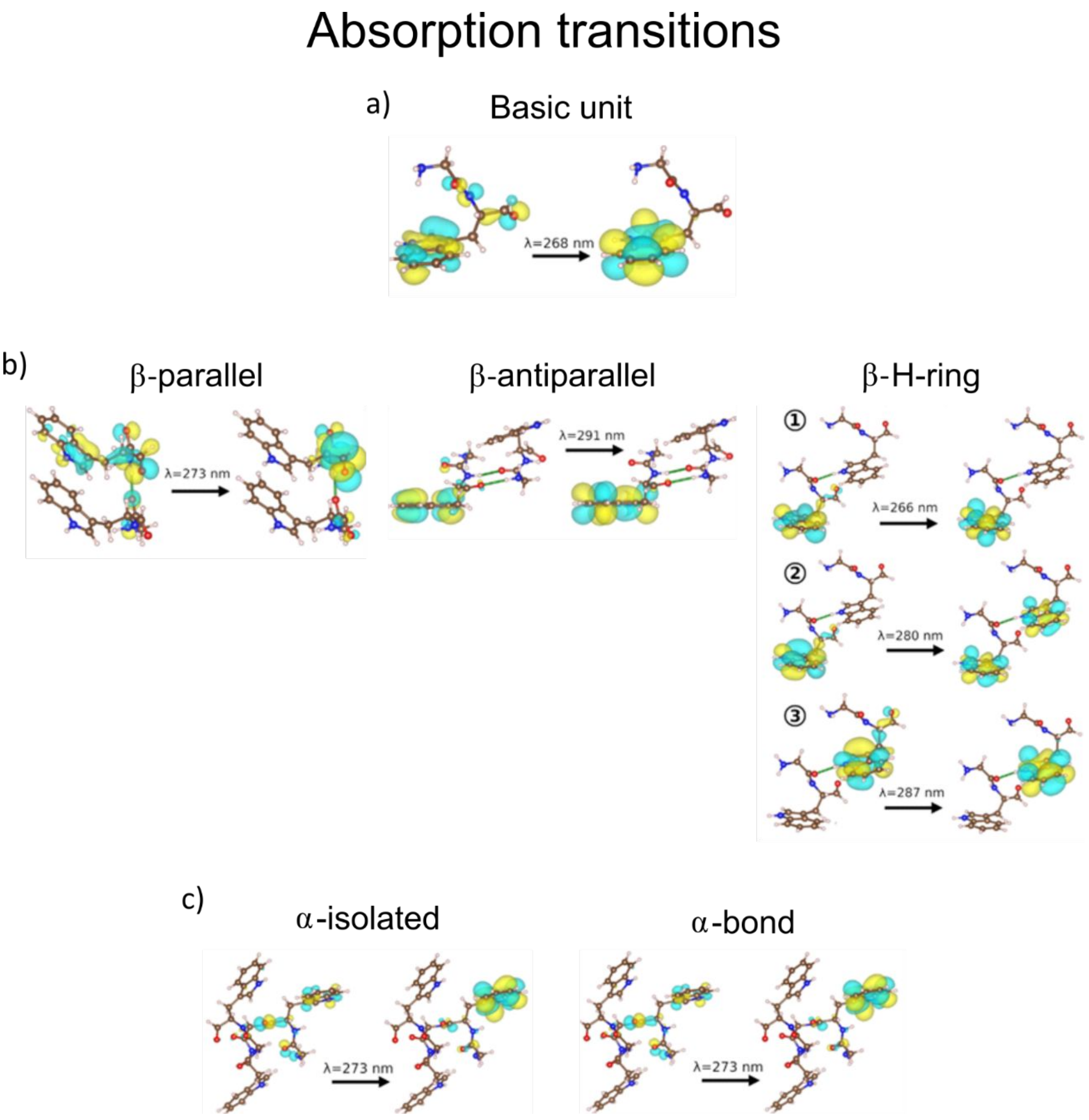

Figure S13. Electron density of the ground and excited states involved in the absorption processes that lead, after structural relaxation, to a relevant fluorescent emission.

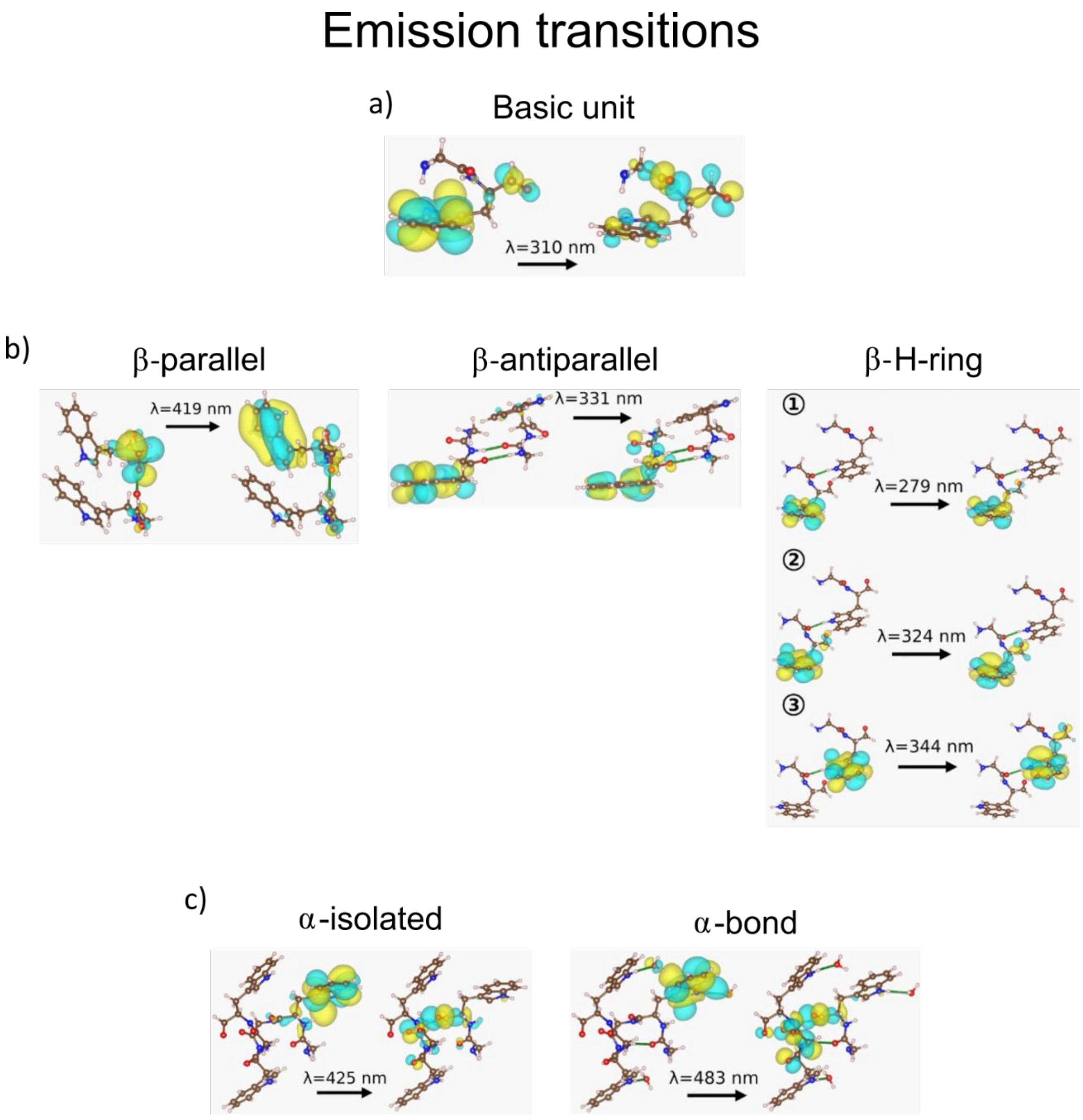

Figure S14: Electron density of the ground state and excited states involved in the emission processes with relevant oscillator strengths.

References

(1) Hammersley, A. European Synchrotron Radiation Facility Internal Report ESRF97HA02T; European Synchrotron Radiation Facility, 1997.
